# Association between meteorological factors, air pollutants and daily hospitalizations of coronary heart disease in rural areas of southern Xinjiang, China

**DOI:** 10.3389/fpubh.2025.1615288

**Published:** 2025-08-21

**Authors:** Xueying Han, Rulin Ma, Jia He, Bo Yang, Silin Chen, Xuelian Wu, Dilimulati Muhetaer, Lanqing Ma, Shijie Shen, Xiaoxue Li, Pengxiang Zuo, Heng Guo

**Affiliations:** Shihezi University School of Medicine, Shihezi, China

**Keywords:** coronary heart disease, meteorological factors, air pollutants, hospitalizations, interaction

## Abstract

**Introduction:**

Meteorological factors and air pollutants are two important factors affecting hospitalisation for coronary heart disease. This study aims to investigate the effects of meteorological factors and air pollutants on the risk of coronary heart disease hospitalisation and their interactions in rural areas with heavy particulate matter pollution at the edge of the desert in southern Xinjiang.

**Methods:**

In this study, patients with coronary heart disease who were hospitalized in Tangyi Town, Tumushuke City, Xinjiang Province, were selected as the study subjects, and the lagged effects of meteorological factors and air pollutants on the risk of coronary heart disease hospitalisation and their interactions were analysed by combining the distributional lag nonlinear model and the quasi-Poisson regression model.

**Results:**

The results showed that the associations between meteorological factors and air pollutant concentrations with the risk of coronary heart disease hospitalisation both showed non-linear and lagged effects. There was an antagonistic effect between mean daily temperature and PM_2.5_ and PM_10_ on the effect of coronary heart disease hospitalisation, with RERIs of −0.73 (95% CI: −2.63, −0.04), and −1.14 (95% CI: −1.93, −0.60), respectively. The relative risk of coronary heart disease hospitalisation in the low-temperature, high PM10 concentration environment was 1.53 (95% CI: 1.09, 2.13). The risk of hospitalization for coronary heart disease is increased by 30 and 19% in environments with low humidity and high PM_2.5_ and PM_10_ concentrations, respectively. There are also interactions between particulate matter and gaseous pollutants and between different gaseous pollutants.

**Discussion:**

This study suggests the need to necessity of management of multiple air pollutants and response to climate change, as well as the importance of implementing targeted preventive and control measures by the relevant authorities in according to meteorological and air pollution conditions, which can effectively reduce the hospitalization rate of patients with coronary heart disease.

## 1 Introduction

Coronary heart disease (CHD) is a cardiovascular disease caused by coronary atherosclerosis leading to coronary artery stenosis or blockage, which ultimately causes myocardial ischemia or hypoxia (1). According to the Global Burden of Disease (GBD) statistics, CHD is the leading cause of death in the world, with 9 million deaths worldwide (2, 3). The China Cardiovascular Health and Disease Report shows that the number of people suffering from CHD in China is 11.39 million, making it one of the main causes of death among residents. Meteorological factors and air pollutants are two important factors affecting CHD (4). A large number of studies are exploring the environmental factors related to CHD including meteorological factors and air pollutants. For example, a national study in China found that a 10% increase in ambient humidity during the summer months increased the risk of Cardiovascular Diseases (CVD) by 17%; further subgrouping of ambient humidity during the summer months showed a “U” shaped effect, meaning that both dry and wet environments may increase the risk of CVD (5). Miao’s findings suggest that the risk of acute myocardial infarction(AMI) increases progressively with decreasing temperature, especially at extremely cold temperatures (−2°C), an environment in which the cumulative Relative Risk(RR) for AMI at a lag of 30 days is 4.66 (1.76, 12.30), but a peak in RR was observed at approximately 24°C as the temperature increased (6). Wang’s study found that the risk of CVD hospitalization increased by 2.3 and 0.8% for each 10 μg/m^3^ increase in PM_2.5_ and PM_10_ concentrations, respectively. Both PM_2.5_ and PM_10_ had a cumulative lag effect on the number of CVD hospitalizations, with a 7-day cumulative RR of 1.115 for PM_2.5_ and a 3-day cumulative RR of 1.015 for PM_10_ (7). Li’s study analyzed the effects of various pollutants and meteorological conditions on CHD, and found that for every 10 μg/m^3^ increase in PM_10_, NO_2_, and SO_2_ concentrations, the risk of death from CHD increased by 0.4, 1.1, and 1.5%, respectively. Heat wave and cold wave events increased the risk of CHD death by 20.2 and 19.9%, respectively. In addition, there was a synergistic effect between heat waves and PM_10_, which increased the risk of CHD death by an additional 37% (8). These studies have demonstrated that the effects of meteorological factors and air pollutants on CHD have not only acute effects, but also non-linear and lagged effects, and that the effects of different meteorological factors and air pollutants vary in different areas. Our study area is located at the edge of the Tarim Basin in southwestern Xinjiang, where the diurnal temperature difference is large and severely affected by particulate matter. Currently, there are few studies on the effects of meteorological factors and air pollutants on CHD in rural areas of southern Xinjiang. Previous studies have mostly used generalized additive model, with little analysis of lagged effects. Compared with generalized additive model, distributed lag nonlinear models (DLNM) can better utilize cross-basis functions to add lag dimensions to the exposure-effect relationship and simultaneously evaluate the lag effects and nonlinear effects of exposure factors (9). Therefore, this study used DLNM to assess the effects of short-term exposure to meteorological factors and air pollutants on the risk of CHD hospitalization in rural areas of southern Xinjiang, and to provide a scientific basis for further assessment of the health effects of meteorological factors and air pollutants.

## 2 Methods and materials

### 2.1 Data source

**Figure 1:**
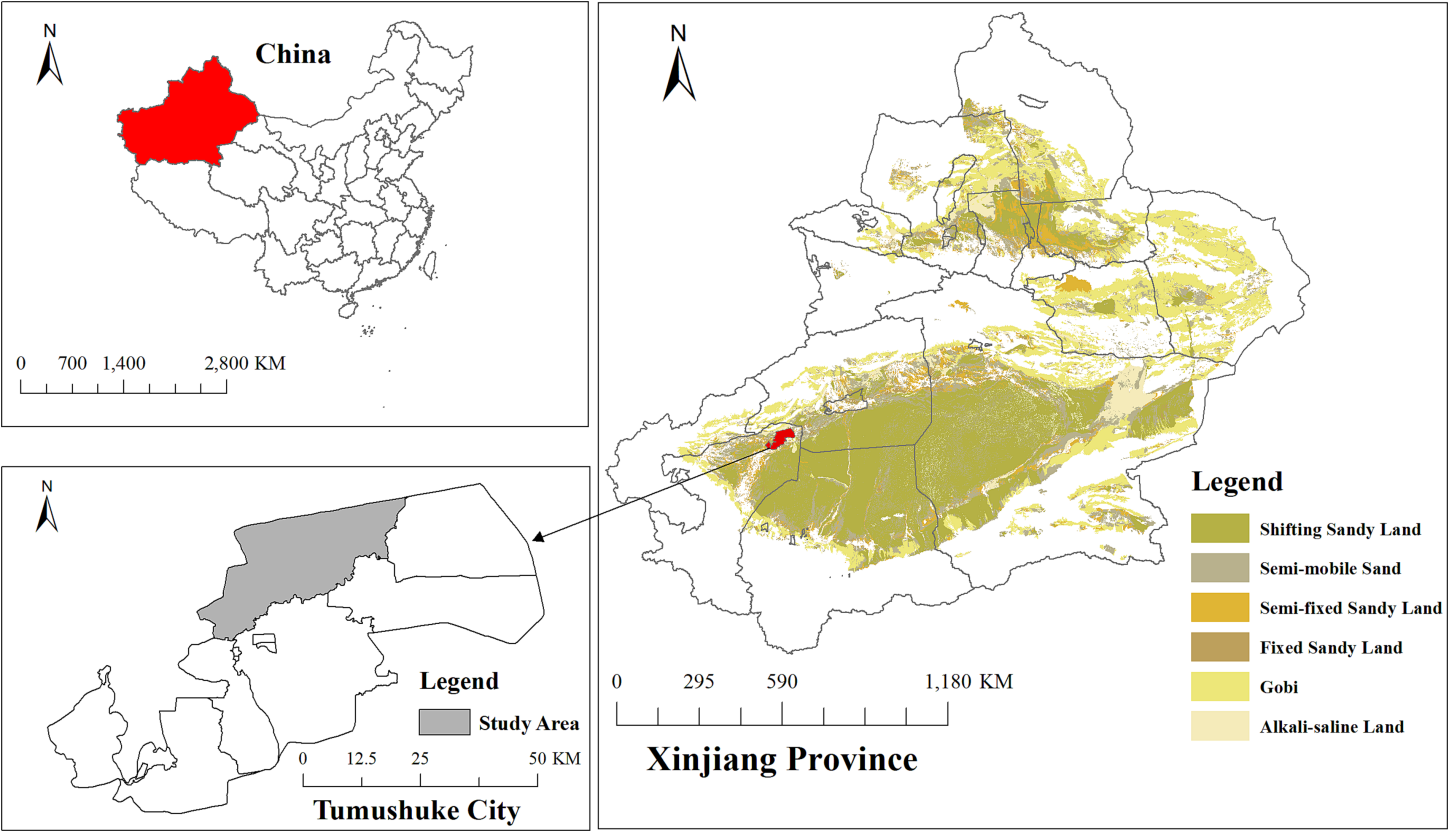
Geographic location of Tangyi Township, Tumushuke City, Xinjiang Province, China. Data source for desert distribution layer: Wang et al. (44) 1:100,000 desert dataset, National Tibetan Plateau Data Center.

This study obtained daily CHD hospitalization case data from January 1, 2016 to December 31, 2022 in Tangyi Town, Tumushuke City, Xinjiang Province (Figure 1). The case information included hospitalization date, disease diagnosis code (ICD-10), disease diagnosis name, gender and age. The main hospitalization diagnosis was CHD (ICD-10: I25.1), and 882 hospitalized cases with a disease diagnosis of CHD were included in the analysis.

Pollutant data were obtained based on the China Big Data Seamless Ground particulate matter (PM_2.5_, PM_10_), nitrogen dioxide (NO_2_), sulfur dioxide (SO_2_) dataset and China Seamless Ground Maximum 8-h Sliding Average Ozone (O_3__8h) dataset obtained from Wei (10–14); meteorological data were obtained based on the Third Pole Region Temperature, Precipitation, Specific Humidity, Wind Speed, and Air Pressure dataset obtained from Yang (15). All of the above meteorological and pollutant data are open access. This study began downloading these data on November 30, 2024. ArcGis 10.8 was utilized to convert the above data into raster format data and to extract meteorological and pollutant concentration daily average data for Tangyi Town, Tumushuke City, Xinjiang Province, according to the administrative division area.

The study was approved by the Ethics Review Committee of the First Affiliated Hospital of Shihezi University School of Medicine (shz2010LL01). All experimental protocols involving human data were in accordance with the Declaration of Helsinki.

### 2.2 Statistical analysis

The statistical descriptions of daily mean temperature, relative humidity, and air pollutant concentrations (PM_2.5_, PM_10_, NO_2_, SO_2_, O_3_) were performed using a variety of methods, such as mean and standard deviation, percentile (P_25_, P_50_, P_75_), maximum versus minimum, and the number of days and multiples of pollutant concentration exceedances. To examine the relationship between meteorological variables and pollutant variables, Spearman’s correlation analysis was utilized. Chi-square test was used to statistically analyze the number of CHD hospitalizations by gender, age, and seasonal subgroups. Distributed lag nonlinear models (DLNM) were used to estimate the independent effects of daily mean temperature, relative humidity, and pollutant concentration on CHD, with subgroup analyses based on gender and age. In order to visualize the lagged effects of meteorological factors and pollutant concentrations, we also plotted three-dimensional and contour plots. In order to better understand the interaction effects between variables, we categorized these variables into binary variables by using the point of the minimum relative risk value of the exposure effect curves for mean daily temperature, relative humidity, and pollutants as the threshold point. Quasi-Poisson regression models were then used to estimate the interactions between these variables.

### 2.3 Distributed lag nonlinear model (DLNM)

The DLNM model allows for simultaneous consideration of nonlinear and lagged effects of the exposure variable on the outcome variable by constructing a cross-basis function (9), and quasi-Poisson regression allows for consideration of over-discrete types of data. Hence, the DLNM model for estimating the effects of meteorological factors and pollutant concentrations on CHD hospitalization is as follows:


(1) Yt~Quasi−Poisson(μt)

(2) log(μt)=α1+cb(Xt)+ns(Time,df)+DOWt+Holidayt

In Equation 1, *Yt* represents the number of hospitalized cases of CHD on day *t*; *μt* represents the expected number of CHD hospitalizations on day *t*. In Equation 2, *α_1_* is the intercept; *Xt* represents the variables on day *t*, including mean temperature, relative humidity, PM_2.5_, PM_10_, NO_2_, SO_2_, and O_3_; *cb(X_t_)* was applied to capture the relationship between daily meteorological factors or daily pollutant concentrations (*X_t_*) and the risk of CHD hospitalization. We used the natural spline functions (“ns”) in controlling the confounding of long-time trend. Based on previous literature, the maximum lag period considered in this analysis is 7 days. The degree of freedom (*df*) to control long-term trends is 6 (16). *DOW_t_* and *Holiday_t_* were applied to control for week effects and holiday effects, respectively.

### 2.4 Interaction analysis

The quasi-Poisson regression model was employed to analyze the interaction of meteorological factors with pollutants and between pollutants (16, 17). Prior to the interaction analysis, we conducted a Spearman’s correlation analysis among all meteorological and pollutant variables to assess their interrelationships. Variables with a Spearman’s correlation coefficient greater than 0.8 in absolute value were considered highly correlated and were excluded from the interaction analysis to avoid multicollinearity issues. Daily mean temperature, relative humidity and pollutant variables were categorized based on the exposure-effect curves obtained from the DLNM model. The turning point of the curve or the point of the lowest risk effect value was chosen as the threshold point at which the variables were binary categorized. The lowest risk point corresponds to the lowest level of disease risk on the exposure-effect curve, and using this point as the threshold allows for a better understanding of the interaction effects between variables. For the meteorological and pollutant variables, we define T = 0 if the value of the variable is less than or equal to the value at the threshold point, and T = 1 if it exceeds the threshold point. The interaction model is then constructed as Equations 3, 4:


(3) Yt~Quasi−Poisson(μt)

(4) log(Yt)=α+A+B+A∗B

where *α* is the intercept; When analyzing the interaction between meteorological factors and air pollutants, *A* represents meteorological factors and *B* represents air pollutants; when analyzing the interaction between air pollutants, *A* and *B* represent two different air pollutants, both of which are dichotomous variables; and A*B is the interaction term. The relative risks (*RRs*) associated with *A*, *B* and A*B were obtained from Equation 4, namely, RR_10_, RR_01_ and RR_11_, respectively. From the model, we can obtain the relative risk (RR) values for RR_10_, RR_01_, and RR_11_. These values are used to calculate the interaction relative risk (IRR), as well as the relative excess risk due to interaction (RERI) along with its corresponding 95% confidence interval (CI). These measures are utilized to evaluate the potential interaction effect. The formula for calculating IRR and RERI, along with their 95% CI, is as Equations 5, 6:


(5) $$\mathrm{IRR}={\mathrm{RR}}_{11}/({\mathrm{RR}}_{01}\times {\mathrm{RR}}_{10})$$

(6) $$\text{RERI}={\mathrm{RR}}_{11}-{\mathrm{RR}}_{01}-{\mathrm{RR}}_{10}+1$$

A significant interaction is observed when the 95% CI of the IRR does not include 1 or when the 95% CI of the RERI does not include 0. Specifically, if IRR > 1 or RERI>0, it indicates a synergistic interaction, suggesting that the combined effect of meteorological factor and air pollution or the combined effect between air pollutants is greater than their individual effects alone. On the other hand, if IRR < 1 or RERI < 0, it indicates an antagonistic interaction.

### 2.5 Sensitivity analysis

Sensitivity analysis based on the Bayesian Information Criterion was conducted to determine the df controlling the long-term trends. In addition, the exposure-effect curves of meteorological factors and air pollutants on CHD hospitalization were all univariate analysis results. The exposure response curves of daily meteorological factors and air pollutions to CHD hospitalization, respectively, were univariate analyses. By controlling for confounding analysis, when analyzing one variable (temperature, relative humidity or pollutions), all the others were taken as confounding control to analyze whether the pre- and post-differences were small.

The “dlnm”, “splines” and “mgcv” packages in R software (V.4.4.2) were used to conduct all the analyses.

## 3 Results

### 3.1 Descriptive statistics

A total of 882 cases were hospitalized for CHD from 2016 to 2022. The age < 65 years group had the highest percentage of cases (58.6%). In addition, spring was the season with the highest number of CHD admissions. There were statistically significant differences in the gender, age and season groups of the cases (*p* < 0.05) (Table 1). Spearman’s rank correlation coefficients between meteorological factors and air pollutants are shown in Table 2.

**Table 1:** Description of CHD hospitalization cases, 2016–2022.

Characteristic	Frequency	Rate (%)	*χ^2^*	*p*
Sex group				
Male	411	46.7	8.2	0.004
Female	471	53.4		
Age group				
Age < 65	517	58.6	6.5	0.008
Age ≥ 65	365	41.4		
Season group				
Spring	268	30.4	20.6	<0.001
Summer	221	25.1		
Autumn	195	22.1		
Winter	198	22.4		

**Table 2:** Spearman’s rank correlation coefficients for meteorological factors and air pollutants, 2016–2022.

Variable	Temperature	Relative humidity	O_3_	NO_2_	SO_2_	PM_2.5_	PM_10_
Temperature	1						
Relative humidity	−0.987*	1					
O_3_	0.895*	−0.902*	1				
NO_2_	−0.503*	0.505*	−0.610*	1			
SO_2_	−0.577*	0.552*	−0.457*	0.188*	1		
PM_2.5_	−0.316*	0.277*	−0.390*	0.173*	0.091*	1	
PM_10_	−0.081*	0.034	−0.155*	0.001	−0.159*	0.866*	1

*Represents statistical significance (*p* < 0.05).

PM_2.5_, Particulate matter with aerodynamic diameters ≤ 2.5 μm; PM_10_, Particulate matter with aerodynamic diameters ≤ 10 μm; NO_2_, Nitrogen dioxide; SO_2_, Sulfur dioxide; O_3_, Ozone.

The average concentrations of PM_2.5_, PM_10_, SO_2_, NO_2_, and O_3__8h were measured as 78.8 μg/m^3^, 274.9 μg/m^3^, 11.4 μg/m^3^, 19.6 μg/m^3^, and 100.3 μg/m^3^, respectively. The secondary maximum concentration limits for PM_2.5_, PM_10_, SO_2_, NO_2_, and O_3__8h were 75, 150, 150, 80, and 160 μg/m^3^, respectively. PM_2.5_ and PM_10_ were the pollutants with the most serious exceedance days and maximum exceedance multiples, with maximum exceedance multiples of 8.2 and 16.3, respectively (Table 3).

**Table 3:** Description of meteorological factors and air pollutants, 2016–2022.

Variable	Mean	SD	Min	Median (P_25_, P_75_)	Max	ED	MEF
Meteorological factors							
Mean temperature (°C)	10.8	11.2	12.2	13.2(0.0,20.6)	29.7	–	–
Relative humidity (%)	15.2	9.1	4.6	11.3(7.9,21.7)	46.2	–	–
Air pollution							
PM_2.5_(μg/m^3^)	78.8	67.2	14.1	60.8(43.2,83.8)	613.2	844	8.2
PM_10_(μg/m^3^)	274.9	248.9	40.2	195.6(136.4,293.0)	2444.1	1760	16.3
NO_2_(μg/m^3^)	19.6	7.2	8.3	17.6(14.6,22.4)	54.6	0	0
SO_2_(μg/m^3^)	11.4	5.2	4.8	10.1(7.6,13.3)	45.7	0	0
O_3__8h(μg/m^3^)	100.3	25.3	45.8	102.5(78.9,120.0)	158.0	0	0

Px, the Xth percentile of the data; PM_2.5_, Particulate matter with aerodynamic diameters ≤ 2.5 μm; PM_10_, Particulate matter with aerodynamic diameters ≤ 10 μm; NO_2_, Nitrogen dioxide; SO_2_, Sulfur dioxide; O_3__8h, Maximum 8-h Sliding Average Ozone; SD, standard deviation; ED, Exceedance Days; MEF, Maximum Exceedance Factor.

### 3.2 Association between meteorological factors, air pollutants and the risk of hospitalization for CHD

**Figure 2:**
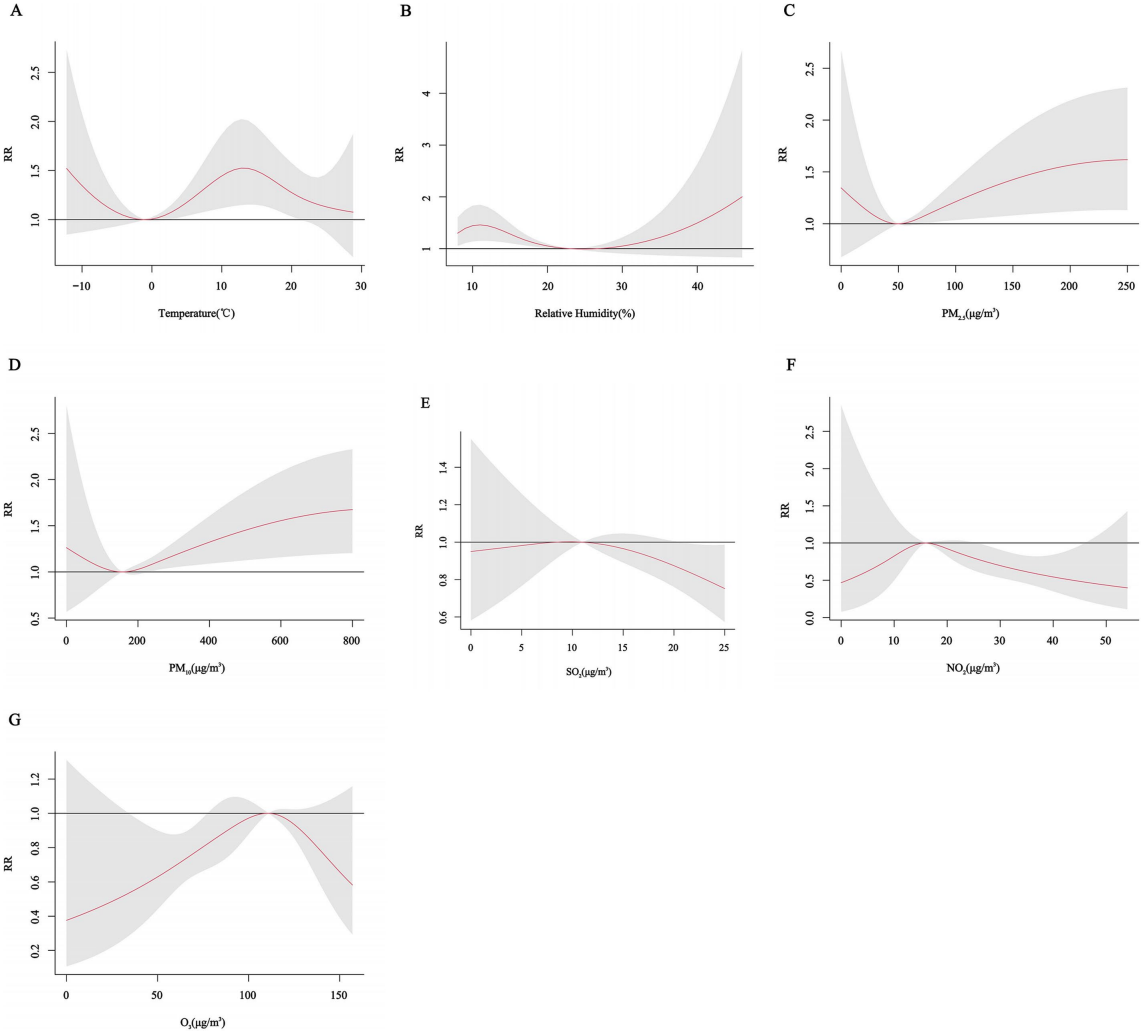
Cumulative exposure-effect curves of the effects of meteorological factors and air pollutants on CHD with a 7-day lag. **(A)** Temperature, **(B)** Relative humidity, **(C)** PM_2.5_: Particulate matter with aerodynamic diameters ≤ 2.5 μm, **(D)** PM_10_: Particulate matter with aerodynamic diameters ≤ 10 μm, **(E)** SO_2_: Sulfur dioxide, **(F)** NO_2_: Nitrogen dioxide, **(G)** O_3_: Ozone.

Figure 2 shows cumulative exposure-effect curves for the effects of meteorological factors and air pollutants on CHD with a 7-day lag. Both lower and higher temperatures and relative humidity increased the risk of CHD hospitalization, with the RR for temperature being greatest at 12.8°C (*RR* = 1.52, 95% CI: 1.14, 2.01) and for relative humidity at 11% (*RR* = 1.46, 95% CI: 1.15, 1.85). The risk of CHD hospitalization increased significantly with higher PM_2.5_, PM_10_ concentrations. The lowest risk points of the exposure-effect curves for mean daily temperature, relative humidity, PM_2.5_, PM_10_, NO_2_, SO_2_, and O_3_ were at −1°C, 24%, 50 μg/m^3^, 156 μg/m^3^, 16 μg/m^3^, 11 μg/m^3^, and 111 μg/m^3^, respectively, and the variables were categorized in a binary fashion at these points.

**Figure 3:**
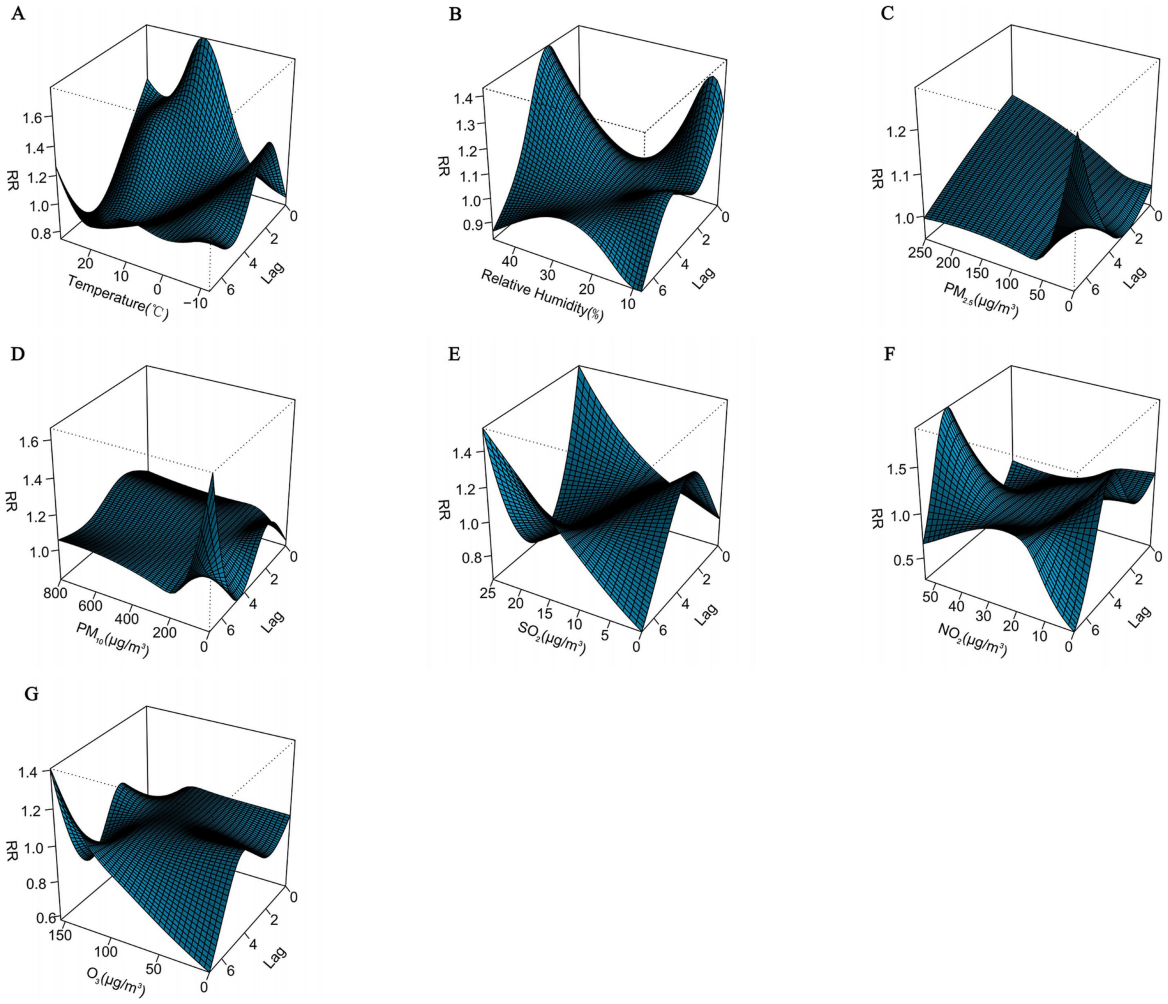
Three-dimensional plots of the association between meteorological factors and air pollutants and CHD with a 7-day lag. **(A)** Temperature, **(B)** Relative humidity, **(C)** PM_2.5_: Particulate matter with aerodynamic diameters ≤ 2.5 μm, **(D)** PM_10_: Particulate matter with aerodynamic diameters ≤ 10 μm, **(E)** SO_2_: Sulfur dioxide, **(F)** NO_2_: Nitrogen dioxide, **(G)** O_3_: Ozone.

The three-dimensional plots showed that the associations of daily mean temperature, relative humidity and pollutant concentrations with CHD all had nonlinear and lagged effects. However, different pollutants had different trends and showed different effects at different lag days (Figure 3).

**Figure 4:**
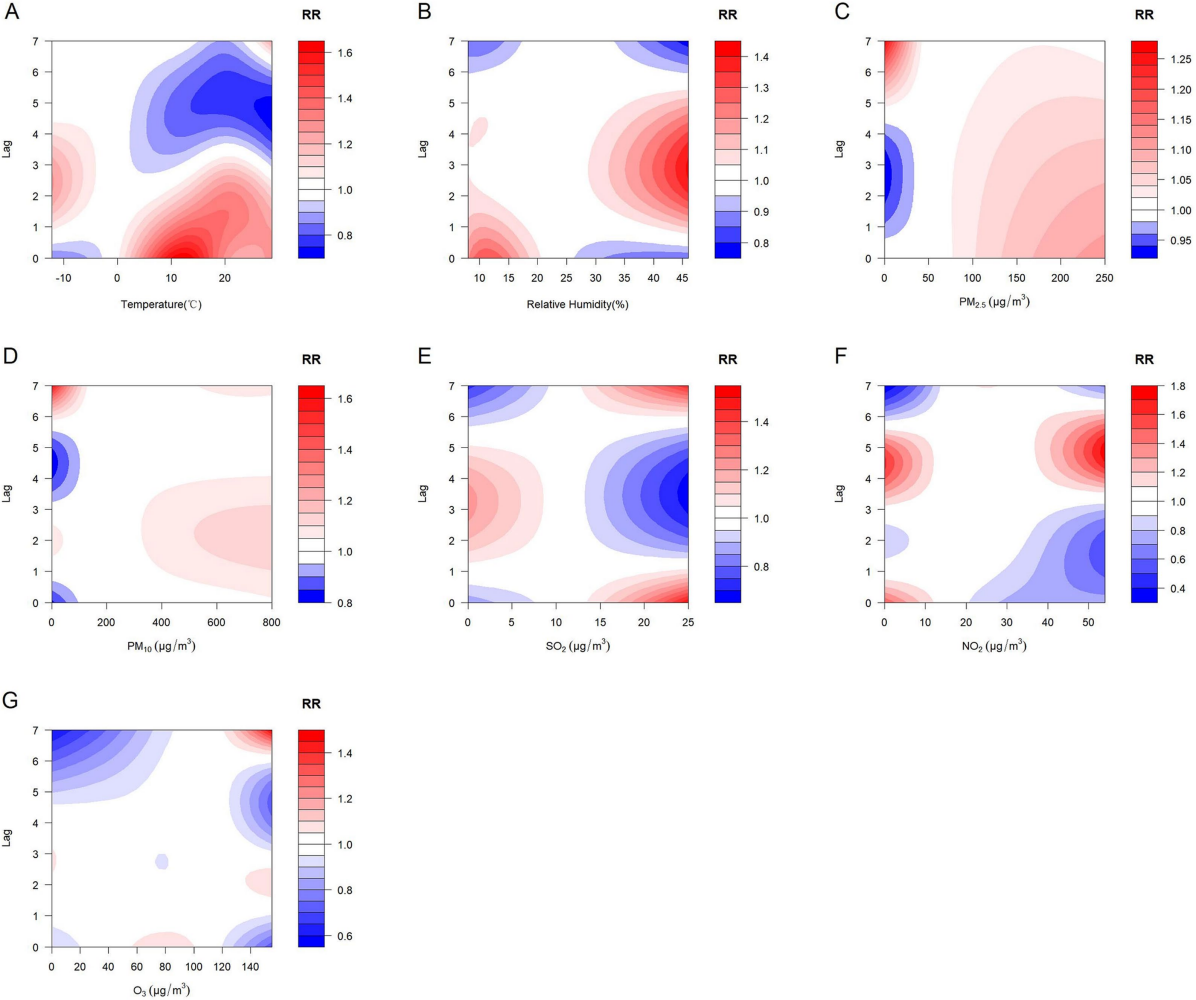
Contour plots of the effects of meteorological factors and air pollutants on CHD with a lag of 7 days. **(A)** Temperature, **(B)** Relative humidity, **(C)** PM_2.5_: Particulate matter with aerodynamic diameters ≤ 2.5 μm, **(D)** PM_10_: Particulate matter with aerodynamic diameters ≤ 10 μm, **(E)** SO_2_: Sulfur dioxide, **(F)** NO_2_: Nitrogen dioxide, **(G)** O_3_: Ozone.

In order to visualize the lagged effects of meteorological factors and air pollutants, we further plotted the contour plots of the effects of meteorological factors and air pollutants on CHD with a lag of 7 days. The contour plots suggest that lower temperature and higher relative humidity may also exhibit varying lag effects on CHD. In addition, different pollutants also have different lag effects (Figure 4).

### 3.3 Subgroup analysis

**Figure 5:**
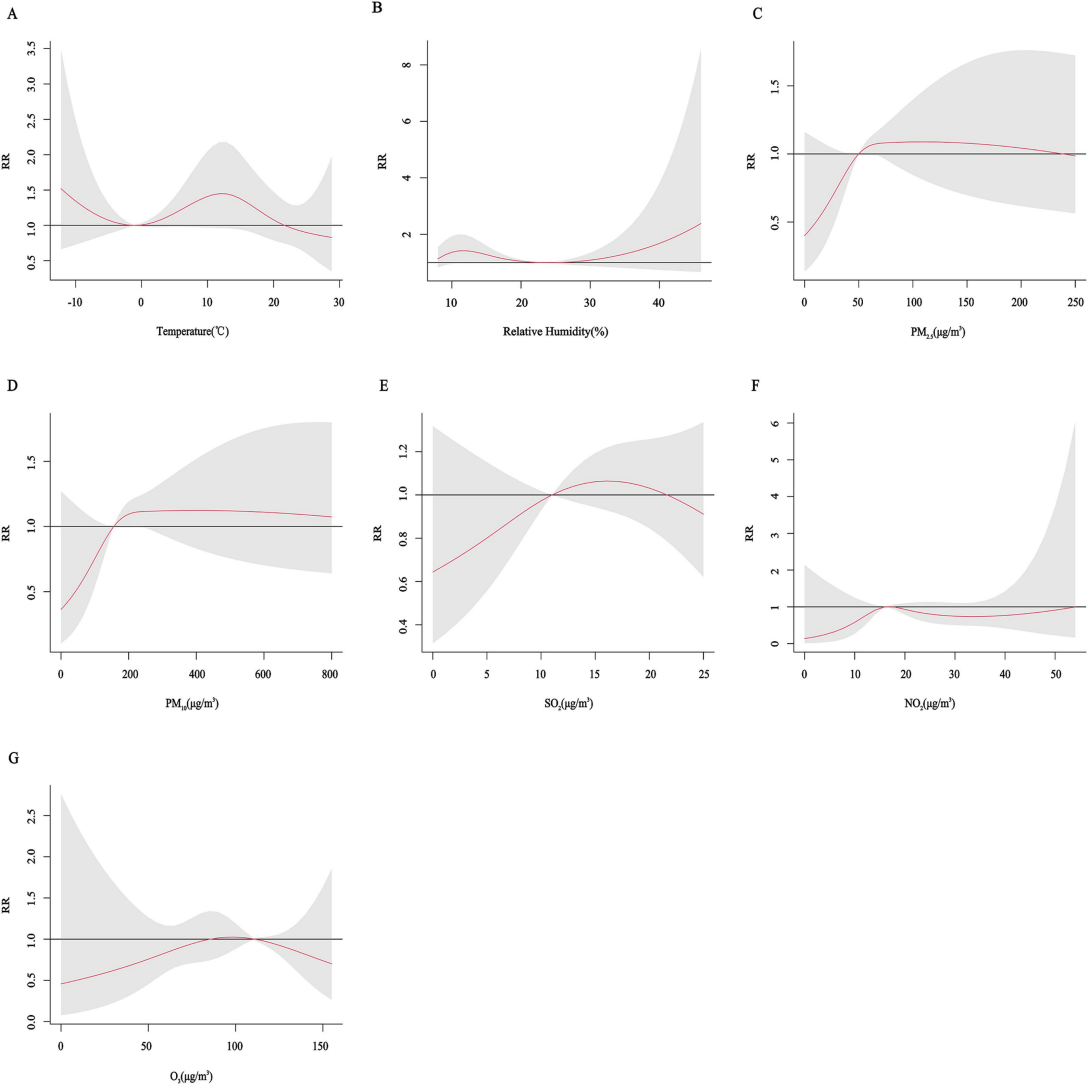
Cumulative exposure-effect curves of the effects of meteorological factors and air pollutants on CHD in male with a 7-day lag. **(A)** Temperature, **(B)** Relative humidity, **(C)** PM_2.5_: Particulate matter with aerodynamic diameters ≤ 2.5 μm, **(D)** PM_10_: Particulate matter with aerodynamic diameters ≤ 10 μm, **(E)** SO_2_: Sulfur dioxide, **(F)** NO_2_: Nitrogen dioxide, **(G)** O_3_: Ozone.

**Figure 6:**
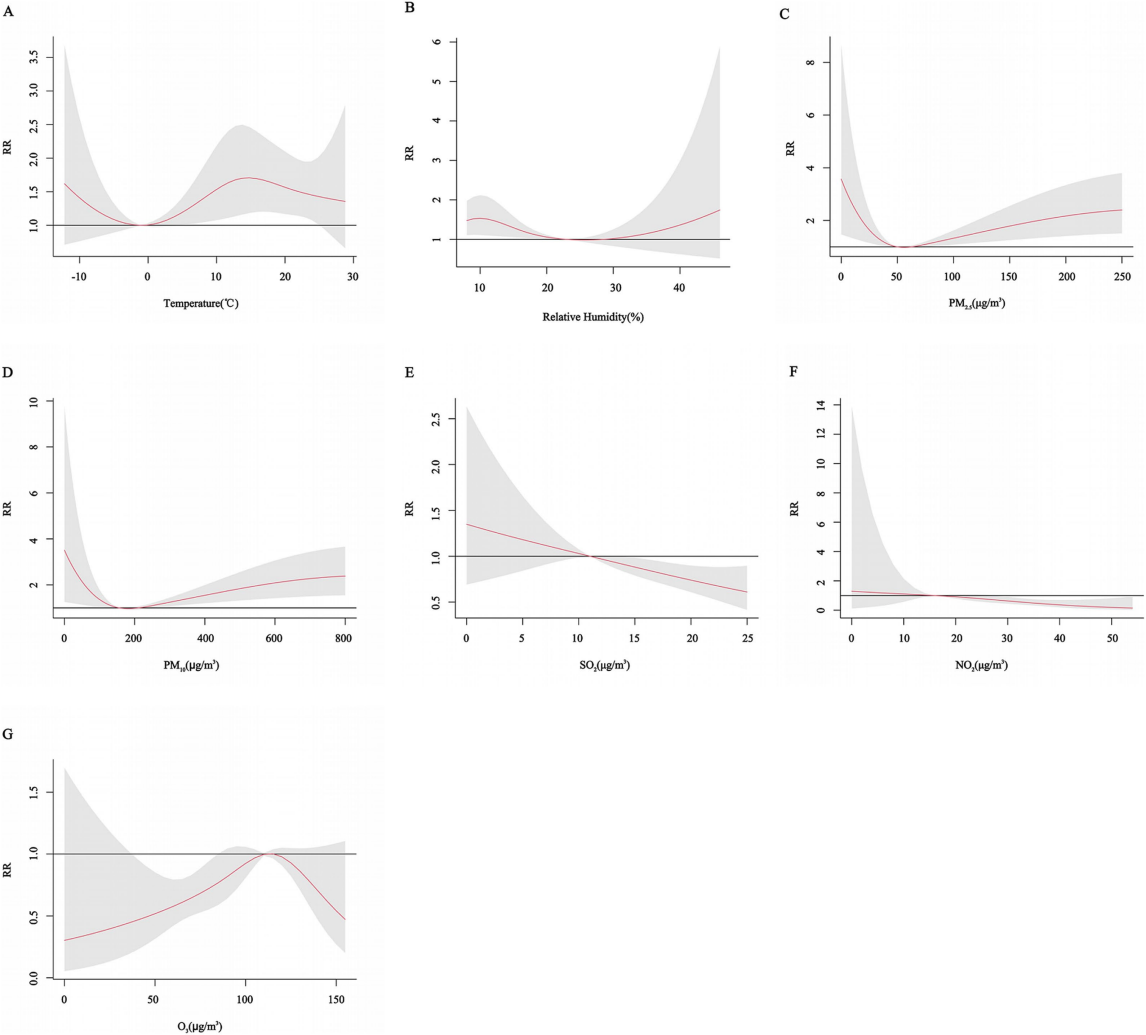
Cumulative exposure-effect curves of the effects of meteorological factors and air pollutants on CHD in female with a 7-day lag. **(A)** Temperature, **(B)** Relative humidity, **(C)** PM_2.5_: Particulate matter with aerodynamic diameters ≤ 2.5μm, **(D)** PM_10_: Particulate matter with aerodynamic diameters ≤ 10 μm, **(E)** SO_2_: Sulfur dioxide, **(F)** NO_2_: Nitrogen dioxide, **(G)** O_3_: Ozone.

Gender subgroup analyses showed that for males, the risk of CHD hospitalization increased with decreasing temperature, peaking at approximately 12°C with a *RR* of 1.46 (95% CI: 0.97, 2.19), but the effect was not statistically significant. The effect on CHD hospitalization tended to increase and then decrease with decreasing relative humidity, peaking at 12% relative humidity with an *RR* of 1.47 (95% CI: 1.03, 2.00), and higher relative humidity did not have a significant effect on hospitalization but tended to increase. The effects of PM_2.5_, PM_10_, SO_2_, NO_2_, and O_3_ on CHD in males increased and then decreased, but none of these effects were significant. In females, the effect of temperature on CHD hospitalization showed a decreasing and then increasing trend, with a non-significant effect at low temperatures, peaking at approximately 15°C with an *RR* of 1.67 (95% CI: 1.16, 2.41), and the effect of relative humidity was similar to that of males, peaking at 10% relative humidity with an *RR* of 1.55 (95% CI: 1.12, 2.14). The effects of PM_2.5_ and PM_10_ concentrations on the risk of CHD hospitalization showed a “U” shaped trend, whereas the effects of SO_2_ and NO_2_ on CHD in females showed a decreasing trend, and the effect of O_3_ showed an inverted “U” shaped trend (Figures 5, 6).

**Figure 7:**
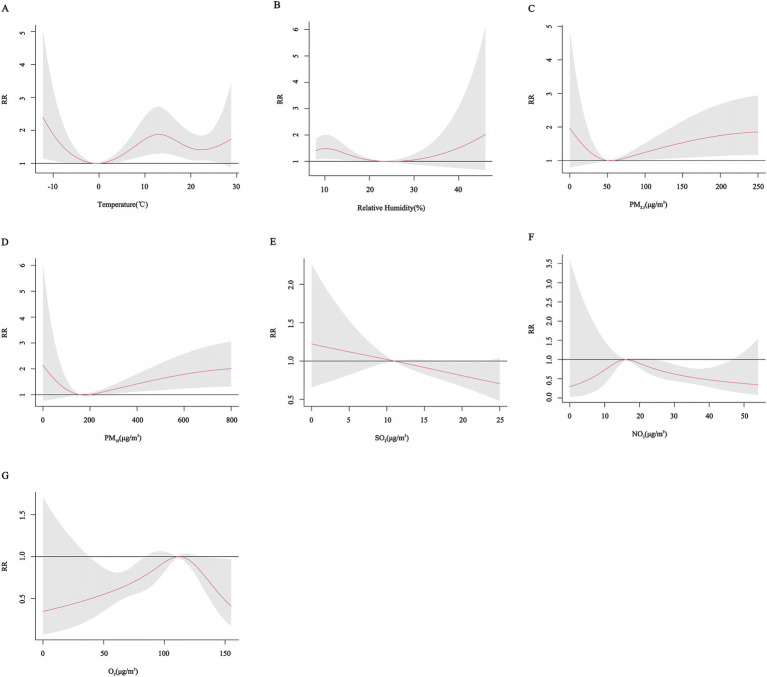
Cumulative exposure-effect curves of the effects of meteorological factors and air pollutants on CHD at age < 65 years with a 7-day lag. **(A)** Temperature, **(B)** Relative humidity, **(C)** PM_2.5_: Particulate matter with aerodynamic diameters ≤ 2.5 μm, **(D)** PM_10_: Particulate matter with aerodynamic diameters ≤ 10 μm, **(E)** SO_2_: Sulfur dioxide, **(F)** NO_2_: Nitrogen dioxide, **(G)** O_3_: Ozone.

**Figure 8:**
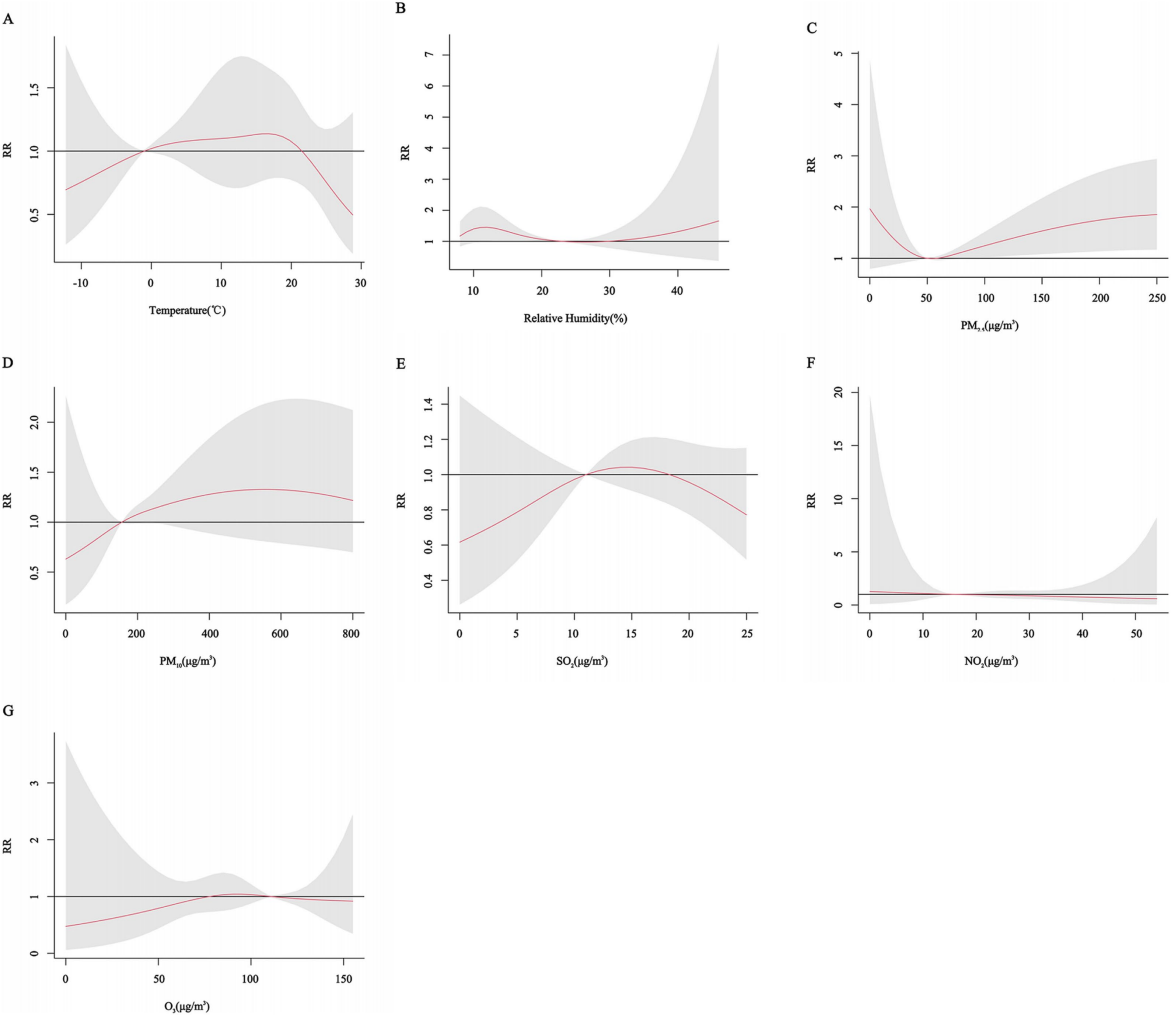
Cumulative exposure-effect curves of the effects of meteorological factors and air pollutants on CHD at age ≥ 65 years with a 7-day lag. **(A)** Temperature, **(B)** Relative humidity, **(C)** PM_2.5_: Particulate matter with aerodynamic diameters ≤ 2.5μm, **(D)** PM_10_: Particulate matter with aerodynamic diameters ≤ 10μm, **(E)** SO_2_: Sulfur dioxide, **(F)** NO_2_: Nitrogen dioxide, **(G)** O_3_: Ozone.

Age subgroup analyses showed a U-shaped trend in the effect of PM_2.5_ concentration and relative humidity on the risk of CHD hospitalization in all age groups. The effect of lower relative humidity on the risk of CHD hospitalization peaked at 12% for all age groups. For the lower age groups (age < 65 years), different trends were observed for different pollutants, with a “U” shaped effect for PM_10_, with the risk of CHD hospitalization gradually increasing with increasing PM_10_ concentrations at higher concentrations, but not significantly at lower concentrations. The effect of temperature on CHD showed a similar U-shaped trend, with an increased risk of CHD hospitalization at both lower (approximately −10°C) and intermediate temperatures (10°C-20°C) and a peak *RR* at 13°C. The effect of SO_2_ showed a gradual decrease with decreasing concentration. The effects of NO_2_ and O_3_ on CHD showed an inverted “U” shaped trend. In the older age group (age ≥ 65 years), the effects of the other variables were not significant, except for the change in PM_2.5_ concentration, which had a significant effect on the risk of hospitalization for CHD. However, it is worth noting that the risk of hospitalization for CHD tended to increase at moderate temperatures (10°C-20°C) or with an increase in the concentration of PM_10_ (Figures 7, 8).

The results of the three-dimensional and contour plots of the gender and age subgroup analyses showed that there was a lagged effect of different meteorological factors and air pollutants on the risk of hospitalization for CHD in different gender and age groups (Supplementary Figures S1–S8).

### 3.4 Interaction analysis

Table 4 shows the interactions between meteorological factors and air pollutants, and between individual air pollutants on the risk of CHD hospitalization. There is a significant interaction between temperature and some pollutants, including PM_2.5_, PM_10_ and NO_2_. The corresponding IRRs and 95% CIs were estimated to be 0.53 (95% CI: 0.24, 0.83) and 0.36 (95% CI: 0.27, 0.47) and 0.24 (95% CI: 0.12, 0.35), respectively. The RERIs and 95% CIs were calculated to be −0.73 (95% CI: −2.63, −0.04) and −1.14 (95% CI: −1.93, −0.60) and −1.59 (95% CI: −3.80, −0.59), respectively, indicating an antagonistic effect between temperature and PM_2.5_, PM_10_ and NO_2_. However, we found that the risk of CHD hospitalization was increased only at low temperatures (<−1°C) with high PM_10_ concentrations (≥156 μg/m^3^) (*RR* = 1.53, 95% CI: 1.09, 2.13), and the antagonistic effect between temperature and PM_2.5_ was not significant. There was also an interaction of relative humidity with PM_2.5_, PM_10_ and NO_2_. The corresponding IRRs and 95% CIs were estimated to be 0.83 (95% CI: 0.80, 0.94) and 1.37 (95% CI: 1.27, 1.52) and 1.36 (95% CI: 1.30, 1.69), respectively. The RERIs and 95% CIs were calculated to be −0.24 (95% CI: −0.30, −0.05) and 0.28 (95% CI: 0.26, 0.37) and 0.29 (95% CI: 0.20, 0.46), respectively, which indicated that there was an antagonistic effect of relative humidity on PM_2.5_ and a synergistic effect on PM_10_ and NO_2_. Similarly, we found that the risk of hospitalization for CHD was increased only in environments with low relative humidity and high concentrations of PM_2.5_ (*p* < 0.05). Interaction analysis between air pollutants showed synergistic interactions between O_3_ and NO_2_ and SO_2_, and between SO_2_ and PM_10_. However, the risk of CHD hospitalization was decreased at low O_3_ and high SO_2_ concentrations, with a *RR* of 0.80 (95% CI: 0.66, 0.96). In addition, there was no interaction between NO_2_ and PM_2.5_ and PM_10_, while antagonistic interactions were observed for the remaining pollutants.

**Table 4:** Interaction analysis between meteorological factors and air pollutants on CHD hospitalization.

Variable	Regressors	*RR*(95%CI)	*P*	IRR(95%CI)	RERI(95%CI)
Temperature, PM_2.5_	T = 1, PM_2.5_ = 1	0.95(0.50,1.83)	0.887	0.53(0.24,0.83)	−0.73(−2.63,-0.04)
	T = 0, PM_2.5_ = 1	1.35(0.72,2.55)	0.355		
	T = 1, PM_2.5_ = 0	1.34(0.72,2.50)	0.363		
Temperature, PM_10_	T = 1, PM_10_ = 1	0.77(0.53,1.11)	0.158	0.36(0.27,0.47)	−1.14(−1.93,-0.60)
	T = 0, PM_10_ = 1	1.53(1.09,2.13)	0.014		
	T = 1, PM_10_ = 0	1.38(1.03,1.87)	0.034		
Temperature, NO_2_	T = 1, NO_2_ = 1	0.59(0.33,1.59)	0.077	0.24(0.12,0.35)	−1.59(−3.80,-0.59)
	T = 0, NO_2_ = 1	1.42(0.81,2.49)	0.219		
	T = 1, NO_2_ = 0	1.76(1.02,3.05)	0.043		
Temperature, SO_2_	T = 1, SO_2_ = 1	1.15(0.71,1.85)	0.566	1.45(0.91,2.05)	0.36(−0.02,0.52)
	T = 0, SO_2_ = 1	0.80(0.51,1.26)	0.336		
	T = 1, SO_2_ = 0	0.99(0.65,1.51)	0.959		
Relative humidity, PM_2.5_	RH = 1, PM_2.5_ = 1	0.90(0.48,1.68)	0.738	0.83(0.80,0.94)	−0.24(−0.30,-0.05)
	RH = 0, PM_2.5_ = 1	1.30(1.11,1.52)	0.001		
	RH = 1, PM_2.5_ = 0	0.84(0.46,1.52)	0.563		
Relative humidity, PM_10_	RH = 1, PM_10_ = 1	1.21(0.83,1.76)	0.430	1.37(1.27,1.52)	0.28(0.26,0.37)
	RH = 0, PM_10_ = 1	1.19(1.01,1.40)	0.036		
	RH = 1, PM_10_ = 0	0.74(0.55,1.01)	0.052		
Relative humidity, NO_2_	RH = 1, NO_2_ = 1	1.02(0.57,1.84)	0.937	1.36(1.30,1.69)	0.29(0.20,0.46)
	RH = 0, NO_2_ = 1	0.89(0.67,1.02)	0.169		
	RH = 1, NO_2_ = 0	0.85(0.75,1.05)	0.552		
Relative humidity, SO_2_	RH = 1, SO_2_ = 1	0.89(0.56,1.43)	0.629	1.01(0.93,1.19)	0.12(−0.07,0.18)
	RH = 0, SO_2_ = 1	0.93(0.78,1.11)	0.411		
	RH = 1, SO_2_ = 0	0.95(0.63,1.44)	0.813		
PM_2.5_, NO_2_	PM_2.5_ = 1, NO_2_ = 1	1.06(0.79,1.42)	0.703	1.10(0.94,1.30)	0.06(−0.09,0.20)
	PM_2.5_ = 0, NO_2_ = 1	0.80(0.63,1.02)	0.072		
	PM_2.5_ = 1, NO_2_ = 0	1.20(0.97,1.49)	0.102		
PM_2.5_, SO_2_	PM_2.5_ = 1, SO_2_ = 1	0.90(0.65,1.24)	0.506	0.77(0.67,0.90)	−0.31(−0.51,-0.11)
	PM_2.5_ = 0, SO_2_ = 1	0.87(0.66,1.17)	0.358		
	PM_2.5_ = 1, SO_2_ = 0	1.33(1.11,1.59)	0.003		
PM_2.5_, O_3_	PM_2.5_ = 1, O_3_ = 1	1.05(0.73,1.43)	0.752	0.82(0.70,0.95)	−0.22(−0.56,-0.04)
	PM_2.5_ = 0, O_3_ = 1	1.06(0.83,1.35)	0.752		
	PM_2.5_ = 1, O_3_ = 0	1.22(0.98,1.51)	0.081		
PM_10_, NO_2_	PM_10_ = 1, NO_2_ = 1	1.10(0.82,1.48)	0.512	1.17(0.99,1.37)	0.13(−0.01,0.24)
	PM_10_ = 0, NO_2_ = 1	0.80(0.64,1.02)	0.069		
	PM_10_ = 1, NO_2_ = 0	1.17(0.94,1.46)	0.153		
PM_10_, SO_2_	PM_10_ = 1, SO_2_ = 1	1.20(0.89,1.62)	0.226	1.38(1.20,1.59)	0.29(0.25,0.35)
	PM_10_ = 0, SO_2_ = 1	0.75(0.58,0.96)	0.023		
	PM_10_ = 1, SO_2_ = 0	1.16(0.96,1.41)	0.122		
PM_10_, O_3_	PM_10_ = 1, O_3_ = 1	0.77(0.57,1.03)	0.078	0.44(0.38,0.51)	−0.88(−1.27,-0.56)
	PM_10_ = 0, O_3_ = 1	1.24(0.98,1.56)	0.074		
	PM_10_ = 1, O_3_ = 0	1.41(1.15,1.73)	0.001		
NO_2_, SO_2_	NO_2_ = 1, O_2_ = 1	1.21(0.89,1.64)	0.232	1.83(1.60,2.13)	0.58(0.57,0.61)
	NO_2_ = 0, SO_2_ = 1	0.80(0.62,1.03)	0.080		
	NO_2_ = 1, SO_2_ = 0	0.83(0.68,1.01)	0.055		
NO_2_, O_3_	NO_2_ = 1, O_3_ = 1	1.05(0.78,1.41)	0.751	1.35(1.19,1.52)	0.29(0.23,0.35)
	NO_2_ = 0, O_3_ = 1	0.94(0.76,1.17)	0.564		
	NO_2_ = 1, O_3_ = 0	0.83(0.68,1.01)	0.061		
O_3_, SO_2_	O_3_ = 1, SO_2_ = 1	1.22(0.86,1.73)	0.260	1.73(1.68.1.75)	0.54(0.46,0.69)
	O_3_ = 0, SO_2_ = 1	0.80(0.66,0.96)	0.017		
	O_3_ = 1, SO_2_ = 0	0.89(0.74,1.06)	0.200		

T, Temperature; RH, Relative humidity; PM_2.5_, Particulate matter with aerodynamic diameters ≤ 2.5 μm; PM_10_, Particulate matter with aerodynamic diameters≤10 μm; NO_2_, Nitrogen dioxide; SO_2_, Sulfur dioxide; O_3_, Ozone; *P*, *p* value; RR, Relative risk; IRR, Interaction relative risk; RERI, Relative excess risk of interaction.

### 3.5 Sensitivity analysis

We estimated the BIC and AOE of the model when controlling for different degrees of freedom for the long-term trend. At a df of 6, the BIC and AOE values of the model were more desirable, and thus the model was chosen to control for the long-term trend at a degree of freedom of 6. In addition, the differences between the model before and after model adjustments were smaller when analyzing a single variable (either the meteorological factor or the air pollutant) while controlling for the remaining variables as confounders (Supplementary Figures S9, S10).

## 4 Discussion

This study investigated the individual and interactive effects of meteorological factors and air pollutants on CHD. The DLNM model was used to estimate the associations of meteorological factors and air pollutants with CHD hospitalization. The results showed that meteorological factors and air pollutants had nonlinear and lagged effects on the risk of CHD hospitalization, and the magnitude of this effect varied by gender and age. The interaction results indicated that the risk of CHD hospitalization was increased under conditions of low temperature with high concentrations of PM_10_.

Changes in temperature may be a risk factor for cardiovascular disease development and mortality. The cumulative exposure-effect curves of meteorological factors and CHD hospitalization in this study showed that both high and low temperature environments increased the risk of CHD hospitalization, especially in females and those younger than 65 years. This is consistent with the results of previous studies (18–23). Under high temperature conditions, the body regulates body temperature through sweating, and heat exposure of the skin leads to vasodilation, increased cardiac output, and increased cardiac load, which may affect CHD patients and thus lead to an increase in CHD hospitalization under high temperature (24). The low temperature environment will reduce the body heat dissipation through vasoconstriction, but the blood viscosity of the body will increase in the low temperature environment, and the imbalance between myocardial oxygen supply and oxygen demand will also lead to an increased risk of hospitalization for CHD (25, 26). In addition, it has been shown that inflammatory factors correlate with the development of CHD, and heat waves induce increased levels of inflammatory markers, accelerating atherosclerosis and thrombosis (27). Our study also found that the effect of temperature on the risk of CHD hospitalization was not significant in the exposure-response curves for males and the higher age group. This may be because the exact timing of the lagged effect may vary by subgroup, and the cumulative 7-day time window may not adequately capture the critical time point of the health effects of temperature change, resulting in a nonsignificant overall effect. The lagged effect found in the contour plots results further suggests that the effect of temperature change on the risk of CHD hospitalization may need to be captured by more refined analytical methods.

According to the Global Burden of Disease (GBD) estimates, environmental pollution was responsible for approximately 9 million deaths worldwide in 2019, with more than half of these deaths attributed to CVD (3). The present study found that PM_2.5_ and PM_10_ increase the risk of CHD even when cumulative exposures with a 7-day lag are considered. Numerous studies have shown that PM_2.5_ and PM_10_ can increase the risk of CHD by inducing adverse physiologic responses in the body and thus increasing the risk of CHD (26, 28–30).

However, conclusions regarding the effects of gaseous pollutants on CHD are currently inconsistent. For example, a Canadian study showed that NO_2_ exposure led to an increased number of emergency department visits for CHD with a ER of 5.9% (95% CI: 2.1 to 9.9%) (31). In contrast, Xie’s study found no significant association between NO₂ exposure and the number of coronary emergency department visits, but there was a positive correlation between SO_2_ exposure and the number of coronary emergency department visits, although this correlation was not statistically significant (32). Another study showed that high levels of SO_2_ increased the number of CHD visits with a ER of 5.02% (95% CI: 2.23–7.88%), whereas O_3_ exposure was negatively correlated with the number of visits (33). Lin’s study, on the other hand, found that O_3_ exposure increased the number of CHD visits (34).

The cumulative exposure curves in this study showed that NO_2_, SO_2_ and O_3_ had a protective effect on CHD. This may be due to the fact that the present study area is located in southwestern Xinjiang, China, where the levels of these pollutants are well below the national standard limits. In addition, the contour plots in this study showed that there was a lagged effect of NO_2_, SO_2_, and O_3_ on the risk of hospitalization for CHD, and this lagged effect was observed in different gender and age groups, while the cumulative exposure curves failed to capture the critical time points, which may have masked part of the effect. However, the specific mechanisms underlying the effects of various air pollutants on CHD are still not fully understood. Future studies need to further explore the potential pathways through which air pollutants affect CHD in order to provide a basis for effective public health policies.

Our study found an interaction between meteorological factors and air pollutants through interaction analysis. In low-temperature environments, high concentrations of PM_2.5_ and PM_10_ increased the risk of CHD hospitalization. This may be due to the fact that when the ambient temperature is low, especially when the height of the atmospheric boundary layer is low, air mobility is reduced and PM_2.5_ and PM_10_ are more likely to accumulate in the near-surface layer, resulting in elevated concentrations. This accumulation effect increases the exposure of the population to PM_2.5_ and exacerbates its health hazards (35). In low relative humidity environments, high concentrations of PM_2.5_ and PM_10_ also increase the risk of hospitalization for CHD. These results are consistent with previous studies. For example, Wu’s study noted that high concentrations of PM_2.5_ and PM_10_ increased the risk of CHD hospitalization by 23.9, 23.3%, respectively, under low temperature conditions (25). The effect of pollutants on CHD hospitalisation was more pronounced at high relative humidity than at low relative humidity (36). This interaction between meteorological factors and pollutants may be due to the fact that high humidity increases the solubility of pollutants and the hygroscopicity of particulate matter, thus exacerbating the toxicity of air pollutants (37). In turn, high concentrations of pollutants further exacerbate oxidative stress through direct stimulation of the respiratory tract and cardiovascular system and damage to endothelial cells, which leads to impaired vasodilatory function affecting autonomic regulation and aggravating the burden on the cardiovascular system (8, 37, 38). Our study also found an interaction between pollutants. Exposure to high concentrations of PM_10_ and SO_2_ also increased the risk of CHD hospitalisation. This is consistent with the results of most previous studies (39–41). Both airborne particulate matter and gaseous pollutants induce adverse physiologic responses in the body, and simultaneous exposure to both can exacerbate damage to the body, thus further exacerbating the risk of CHD. It was also found that there was also a synergistic interaction between gaseous pollutants and that concurrent exposure increased the risk of CHD hospitalisation. This is consistent with previous findings (41, 42). In addition to triggering metabolic disorders in the respiratory and cardiovascular systems of the body, combined pollutant exposure can also trigger metabolic disorders such as insulin resistance and dyslipidaemia, which can further exacerbate the risk of cardiovascular disease (43).

This study has some limitations. Firstly, the environmental variables in our study were obtained through monitoring stations and cannot accurately reflect individual exposure levels, resulting in potential bias in the results. Secondly, this study is not a community-based study, and we are studying a population in a rural area of southern Xinjiang. The geographic specificity of the study area and the unique living and dietary habits of this population mean that the results may be influenced by specific environmental or population characteristics, which limits the generalizability of the results and makes it possible that the results may not be directly generalizable to other different communities or populations. Third, this study is essentially an ecological study and cannot confirm the causal relationship between meteorological factors and air pollutants and CHD. Fourth, the small number of hospitalizations included in this study may have had an impact on the statistical reliability of the overall results, especially when the data were further subdivided into subgroups. Future studies with larger samples and in more areas are needed to verify the association between meteorological factors and air pollutants on CHD. Finally, it is worth noting that although the COVID-19 pandemic was covered during the period of this study, we believe that its impact on this study was minimal. Strict control measures, inherently low population density, and predominantly agro-pastoralist production practices in the study area limited its spread. These factors effectively minimized the potential interference of COVID-19-related confounding factors with the study results.

## 5 Conclusion

In this study, the cumulative lagged effects of individual meteorological factors or air pollutants on the risk of CHD hospitalization and the interactions between the variables were simultaneously analyzed using a time-series DLNM model. The results of the study indicate that changes in meteorological conditions and high concentrations of airborne particulate matter may increase the risk of hospitalization for CHD in the heavily particulate-polluted rural areas of southern Xinjiang. Low-temperature or low-humidity environments accompanied by high concentrations of particulate matter may be key to the increased risk of hospitalization for CHD. There are also different interactions between gaseous pollutants and particulate matter. It is worth noting that high concentrations of SO_2_ may increase the risk of hospitalization for CHD even when airborne particulate matter concentrations are low. The results of this study emphasize the importance of relevant departments implementing targeted prevention and control measures based on meteorological and air pollution conditions. By doing so, we can effectively reduce the hospitalization rate among CHD patients and alleviate the burden of this disease.

## Data Availability

The original contributions presented in the study are included in the article/Supplementary material, further inquiries can be directed to the corresponding authors.
