# Supplementary material for: Association between meteorological factors, air pollutants and daily hospitalizations of coronary heart disease in rural areas of southern Xinjiang, China
Source: Front Public Health. 2025 Aug 21;13:1615288. doi: 10.3389/fpubh.2025.1615288 (PMC12408508; doi:10.3389/fpubh.2025.1615288)
Supplement: Supplementary file 2 [file Data_Sheet_2.zip › Supplementary Material Presentation-1/Supplementary Material Presentation-1.docx]

FigS1 Three-dimensional plots of the association between meteorological factors and air pollutants and CHD in male with a 7-day lag. PM_2.5_: Particulate matter with aerodynamic diameters≤2.5μm, PM_10_: Particulate matter with aerodynamic diameters≤10μm, NO_2_: Nitrogen dioxide, SO_2_: Sulfur dioxide, O_3_: Ozone

FigS2 Three-dimensional plots of the association between meteorological factors and air pollutants and CHD in female with a 7-day lag. PM_2.5_: Particulate matter with aerodynamic diameters≤2.5μm, PM_10_: Particulate matter with aerodynamic diameters≤10μm, NO_2_: Nitrogen dioxide, SO_2_: Sulfur dioxide, O_3_: Ozone

FigS3 Three-dimensional plots of the association between meteorological factors and air pollutants and CHD at age <65 years with a 7-day lag. PM_2.5_: Particulate matter with aerodynamic diameters≤2.5μm, PM_10_: Particulate matter with aerodynamic diameters≤10μm, NO_2_: Nitrogen dioxide, SO_2_: Sulfur dioxide, O_3_: Ozone

FigS4 Three-dimensional plots of the association between meteorological factors and air pollutants and CHD at age ≥65 years with a 7-day lag. PM_2.5_: Particulate matter with aerodynamic diameters≤2.5μm, PM_10_: Particulate matter with aerodynamic diameters≤10μm, NO_2_: Nitrogen dioxide, SO_2_: Sulfur dioxide, O_3_: Ozone
