# Supplementary figures and images for: Association between meteorological factors, air pollutants and daily hospitalizations of coronary heart disease in rural areas of southern Xinjiang, China
Source: Front Public Health. 2025 Aug 21;13:1615288. doi: 10.3389/fpubh.2025.1615288 (PMC12408508; doi:10.3389/fpubh.2025.1615288)

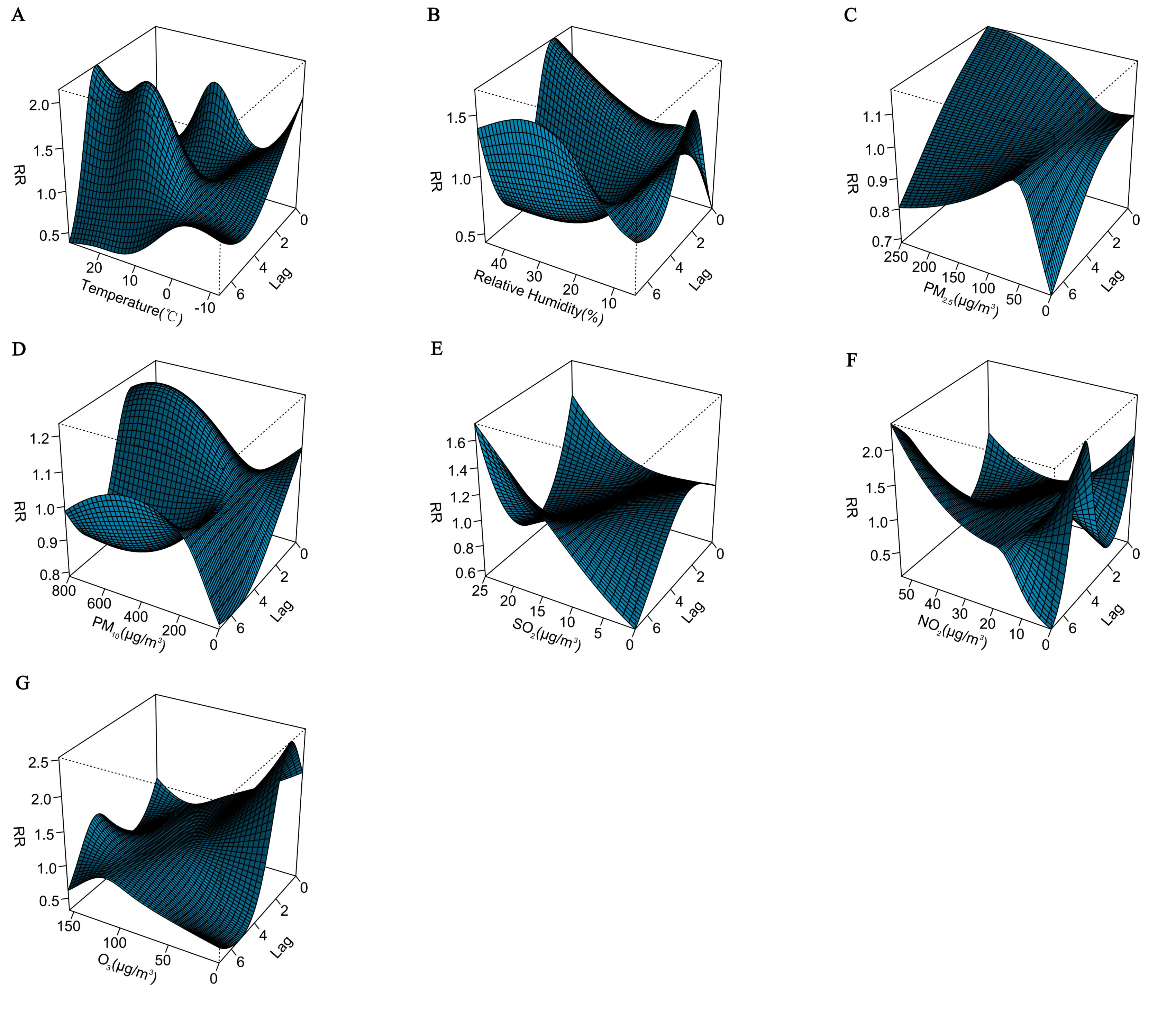

Supplement: Supplementary file 2 [file Data_Sheet_2.zip › Supplementary Material Presentation-1/Figure S1.tif]

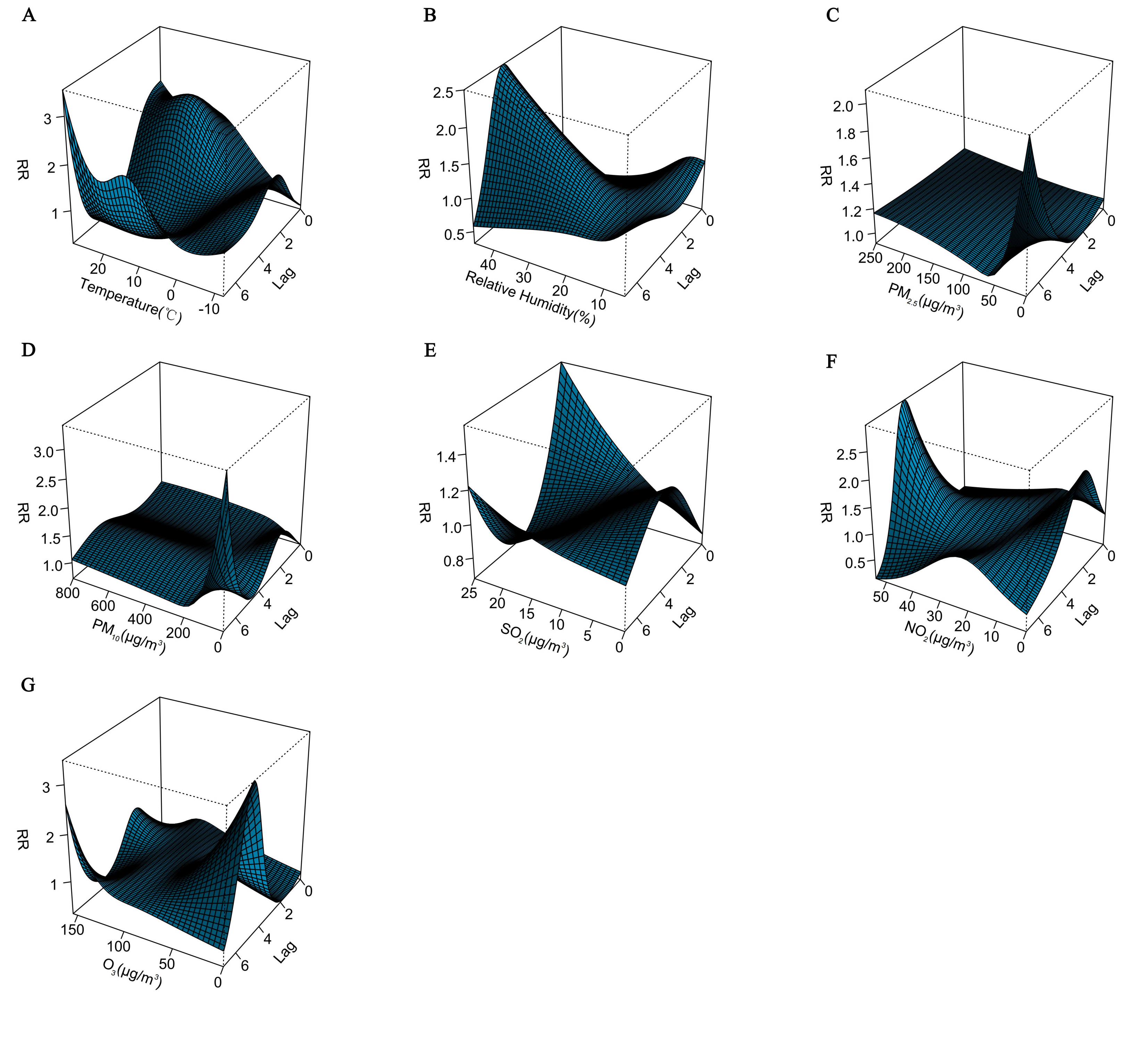

Supplement: Supplementary file 2 [file Data_Sheet_2.zip › Supplementary Material Presentation-1/Figure S2.tif]

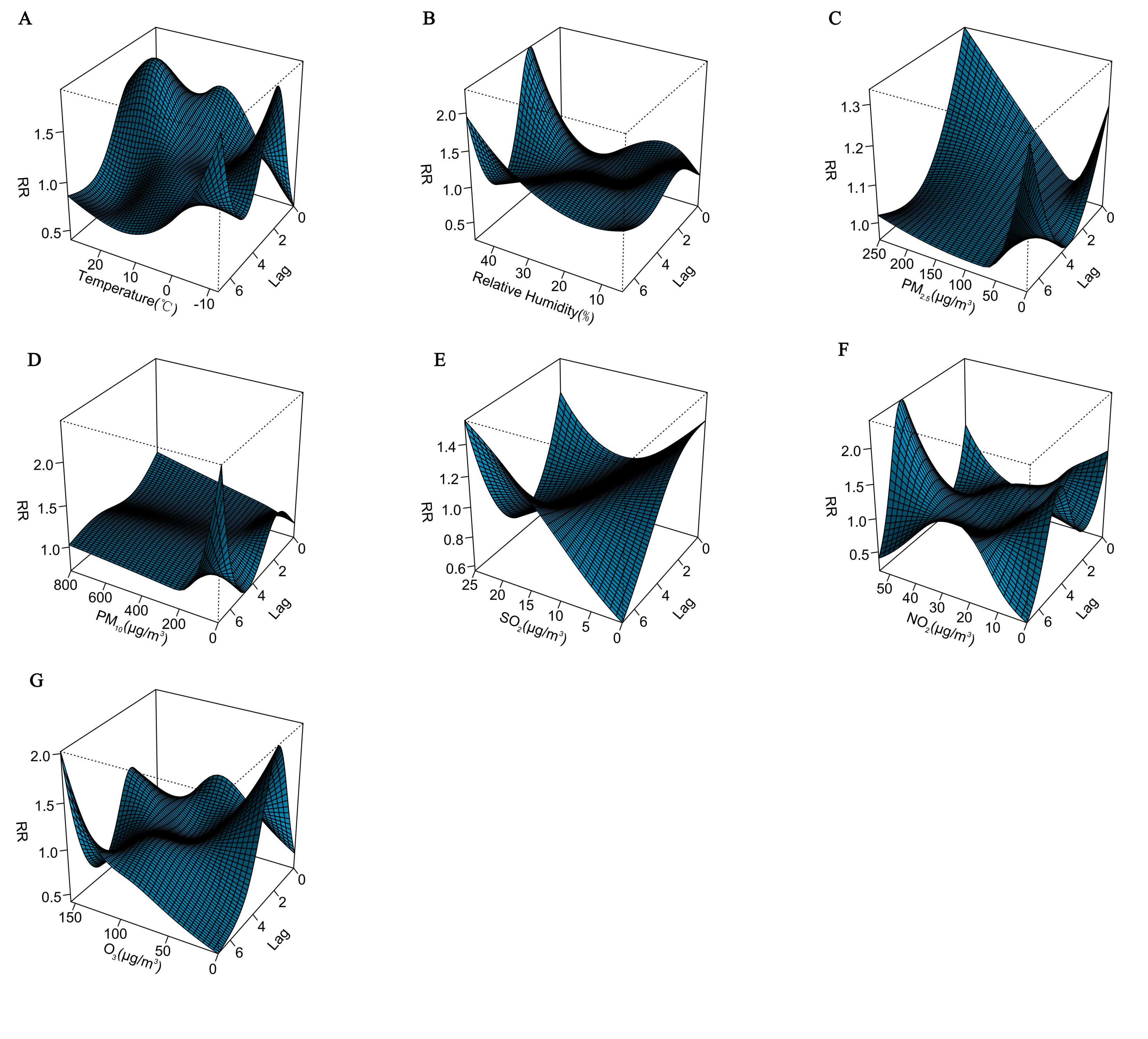

Supplement: Supplementary file 2 [file Data_Sheet_2.zip › Supplementary Material Presentation-1/Figure S3.tif]

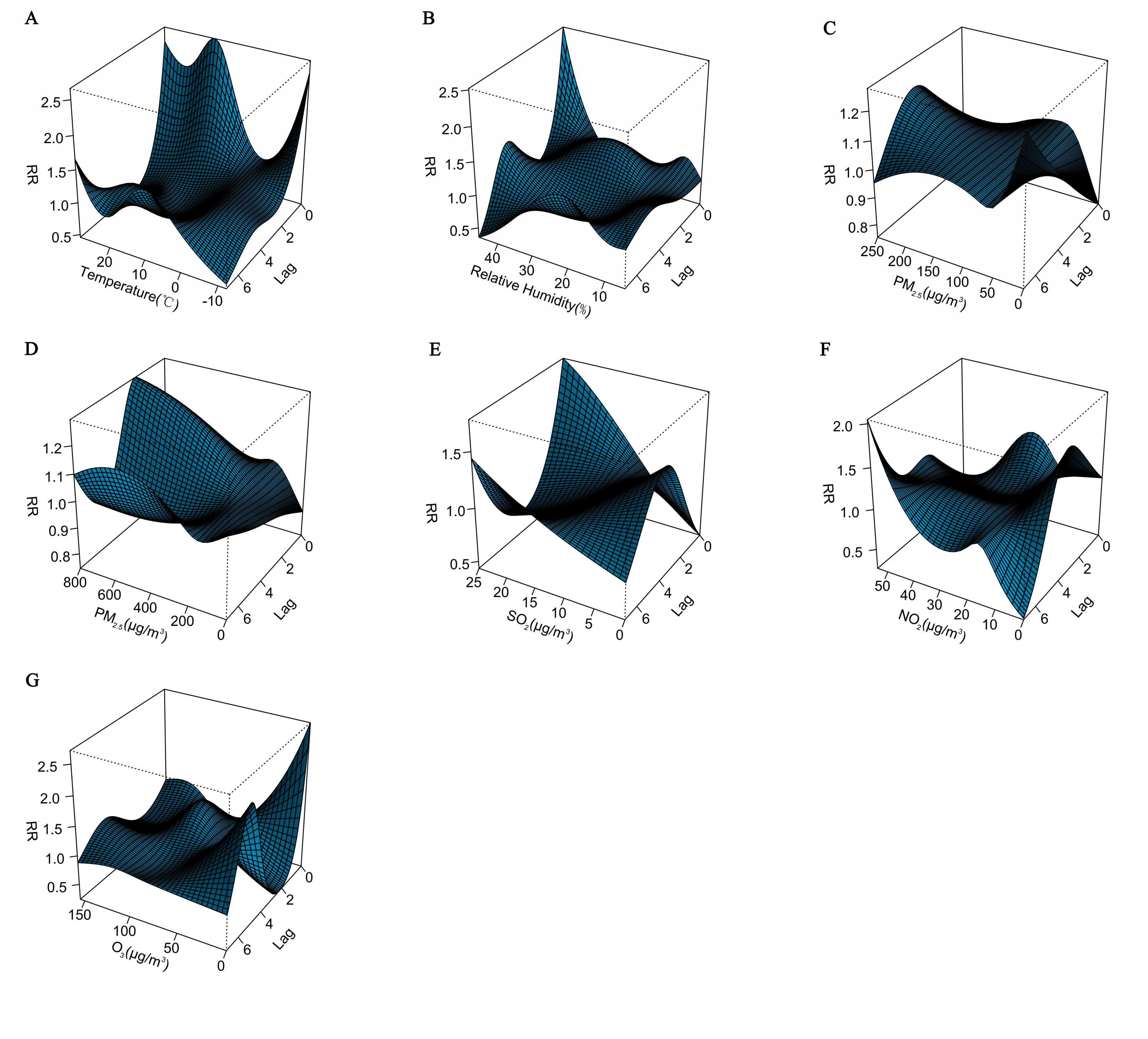

Supplement: Supplementary file 2 [file Data_Sheet_2.zip › Supplementary Material Presentation-1/Figure S4.tif]

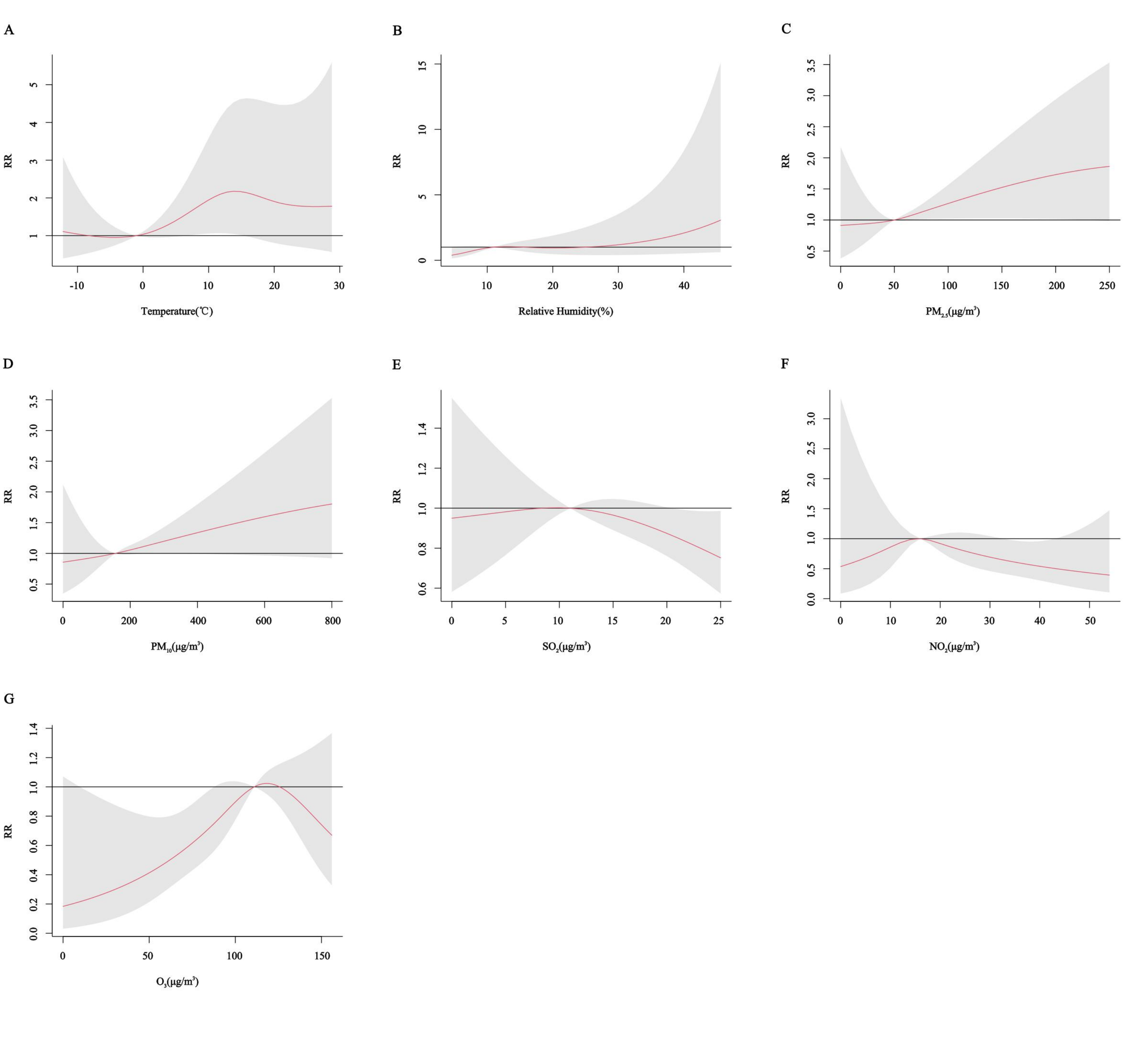

Supplement: Supplementary file 3 [file Data_Sheet_3.zip › Supplementary Material Presentation-2/Figure S10.tif]

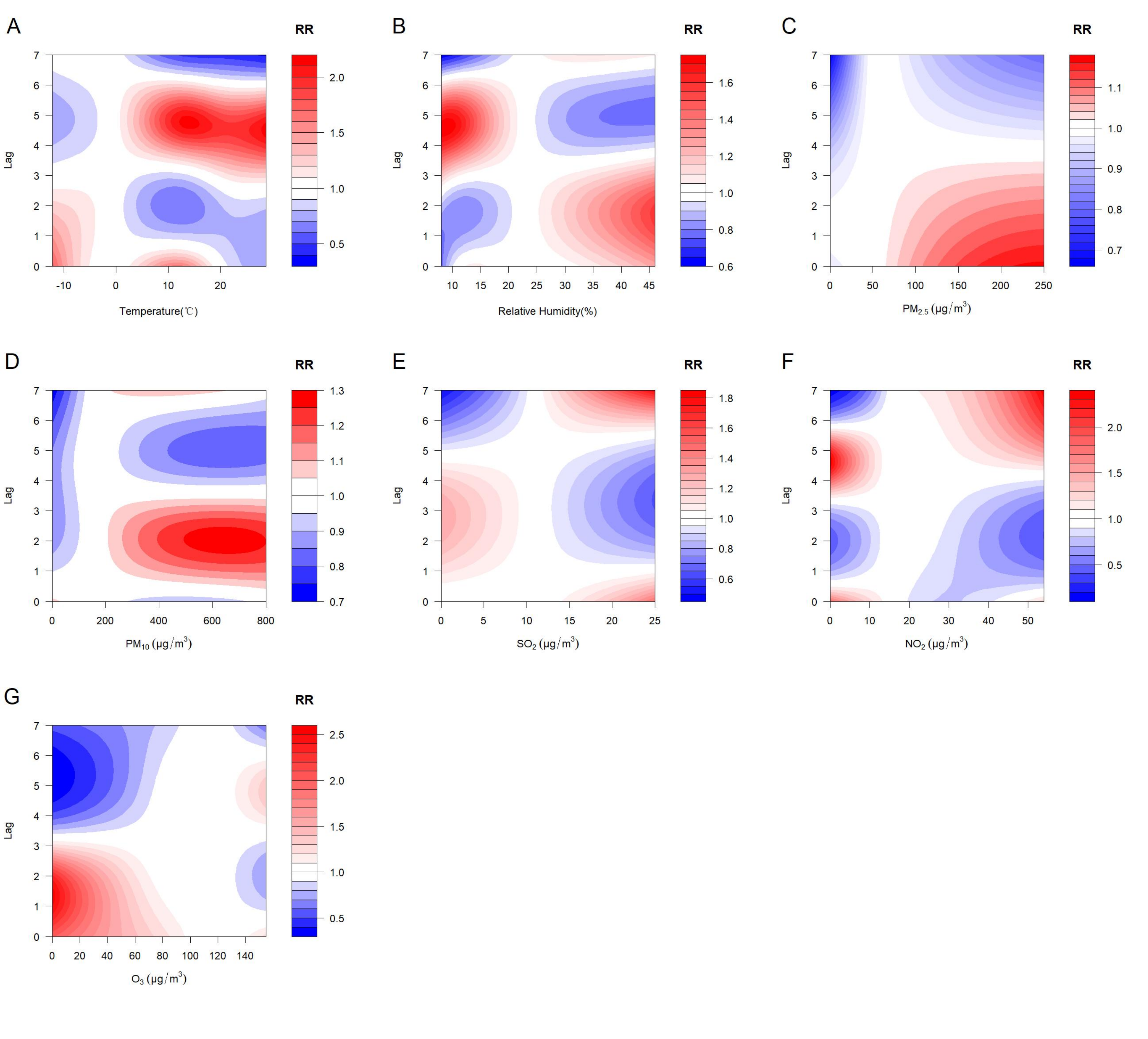

Supplement: Supplementary file 3 [file Data_Sheet_3.zip › Supplementary Material Presentation-2/Figure S5.tif]

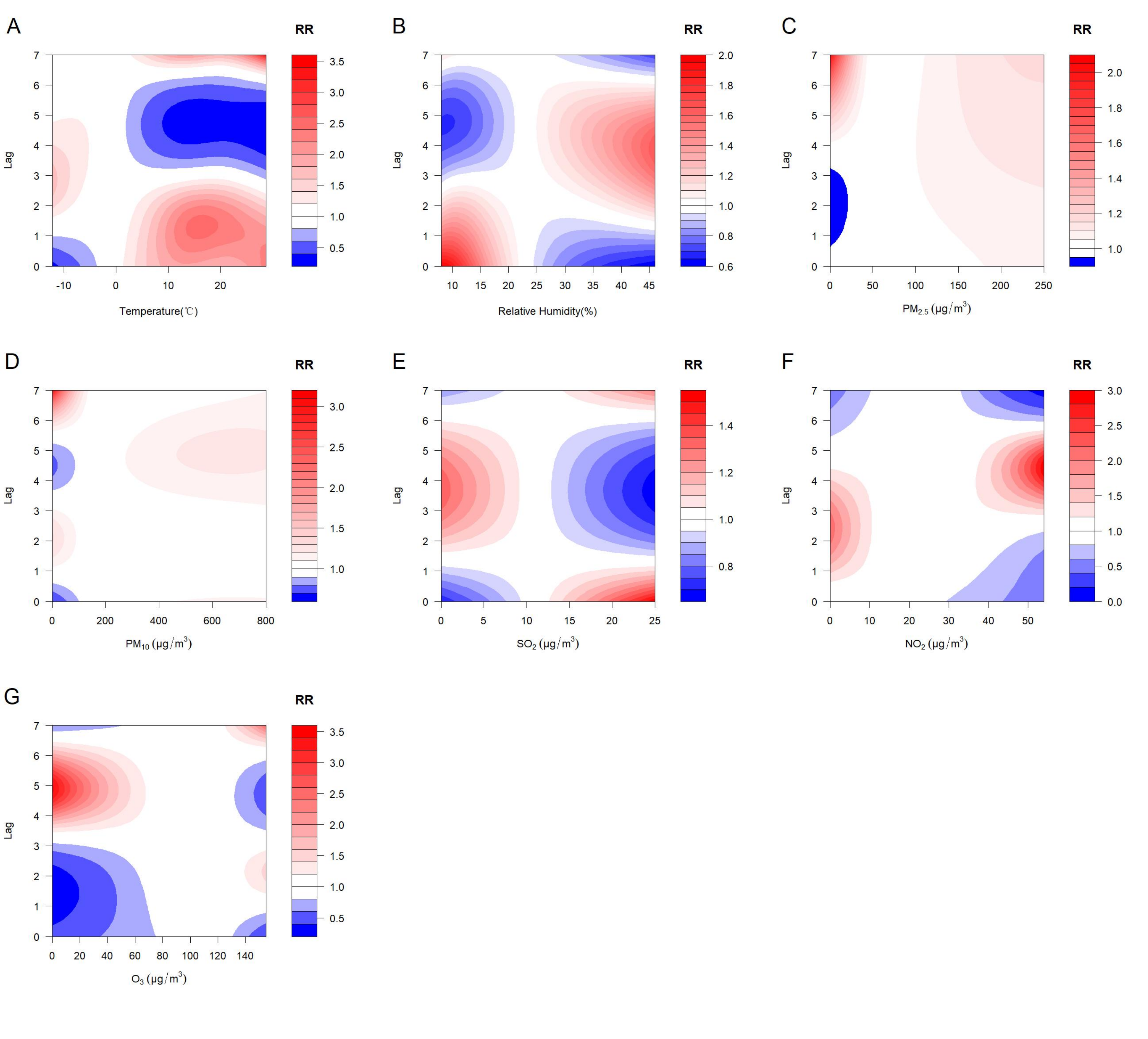

Supplement: Supplementary file 3 [file Data_Sheet_3.zip › Supplementary Material Presentation-2/Figure S6.tif]

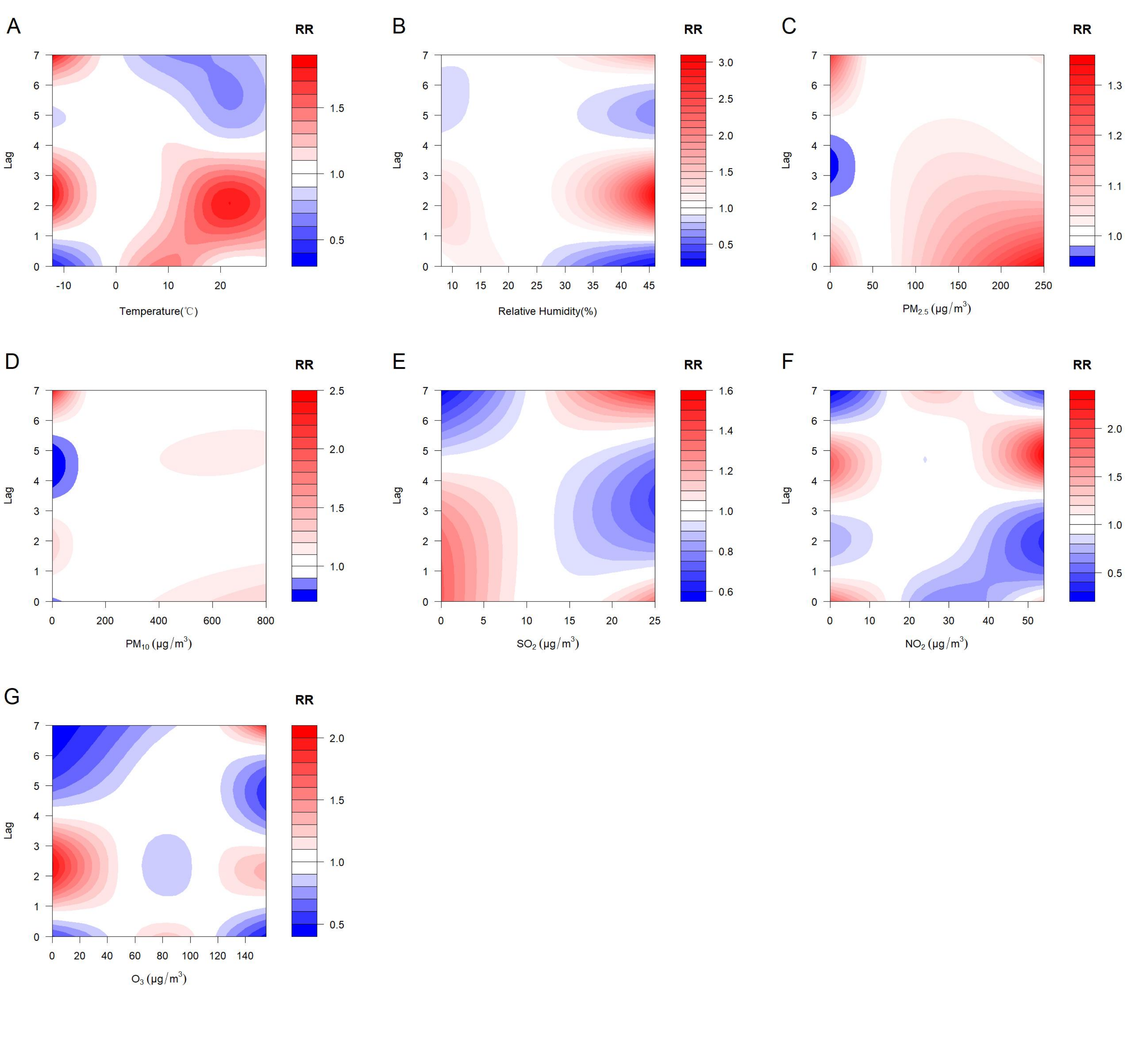

Supplement: Supplementary file 3 [file Data_Sheet_3.zip › Supplementary Material Presentation-2/Figure S7.tif]

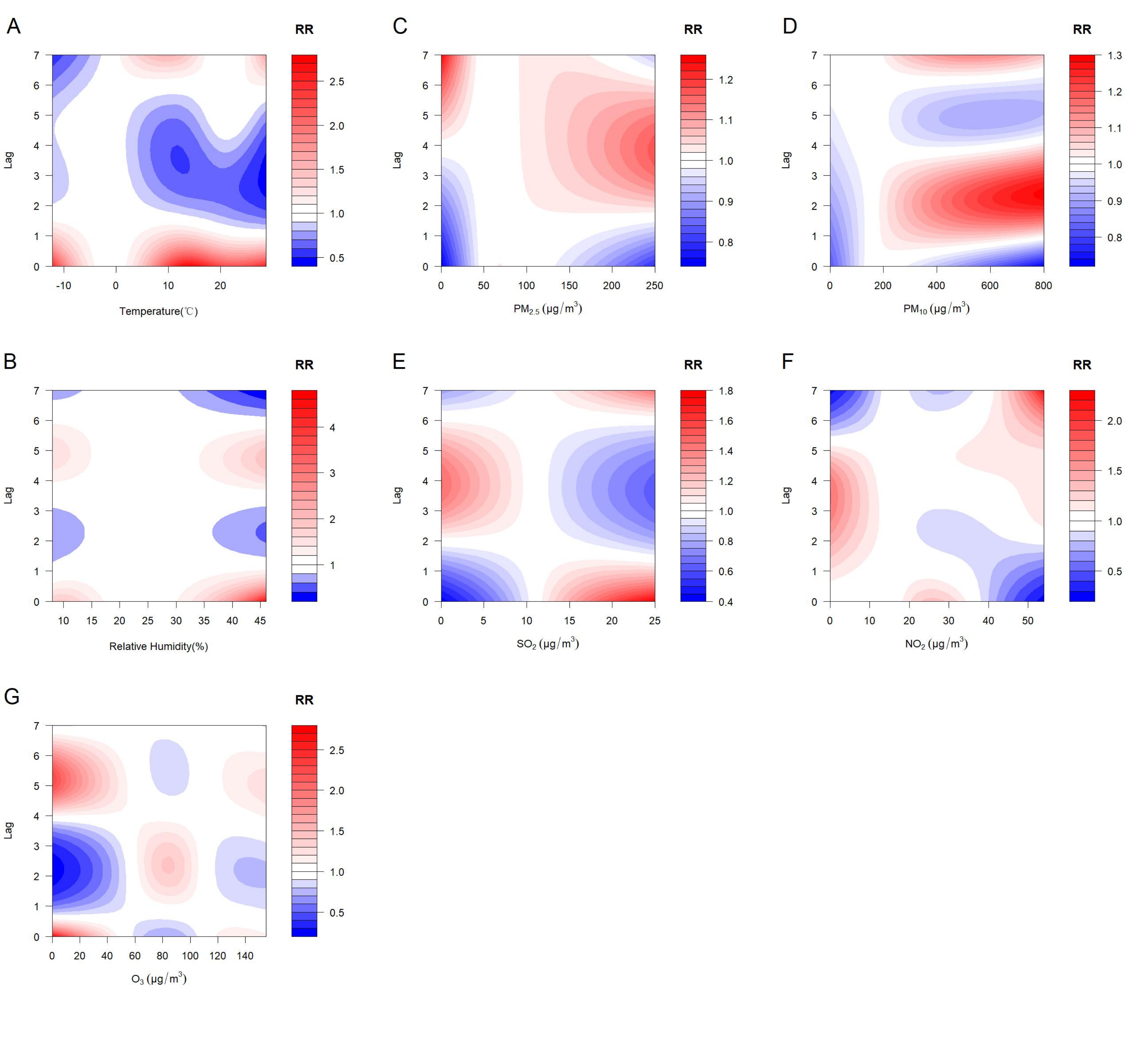

Supplement: Supplementary file 3 [file Data_Sheet_3.zip › Supplementary Material Presentation-2/Figure S8.tif]
