# Supplementary material for: Association between meteorological factors, air pollutants and daily hospitalizations of coronary heart disease in rural areas of southern Xinjiang, China
Source: Front Public Health. 2025 Aug 21;13:1615288. doi: 10.3389/fpubh.2025.1615288 (PMC12408508; doi:10.3389/fpubh.2025.1615288)
Supplement: Supplementary file 3 [file Data_Sheet_3.zip › Supplementary Material Presentation-2/Supplementary Material Presentation.docx]

FigS5 Contour plots of the effects of meteorological factors and air pollutants on CHD in male with a lag of 7 days. PM_2.5_: Particulate matter with aerodynamic diameters≤2.5μm, PM_10_: Particulate matter with aerodynamic diameters≤10μm, NO_2_: Nitrogen dioxide, SO_2_: Sulfur dioxide, O_3_: Ozone

FigS6 Contour plots of the effects of meteorological factors and air pollutants on CHD in female with a lag of 7 days. PM_2.5_: Particulate matter with aerodynamic diameters≤2.5μm, PM_10_: Particulate matter with aerodynamic diameters≤10μm, NO_2_: Nitrogen dioxide, SO_2_: Sulfur dioxide, O_3_: Ozone

FigS7 Contour plots of the effects of meteorological factors and air pollutants on CHD at age <65 years with a lag of 7 days. PM_2.5_: Particulate matter with aerodynamic diameters≤2.5μm, PM_10_: Particulate matter with aerodynamic diameters≤10μm, NO_2_: Nitrogen dioxide, SO_2_: Sulfur dioxide, O_3_: Ozone

FigS8 Contour plots of the effects of meteorological factors and air pollutants on CHD at age ≥65 years with a lag of 7 days. PM_2.5_: Particulate matter with aerodynamic diameters≤2.5μm, PM_10_: Particulate matter with aerodynamic diameters≤10μm, NO_2_: Nitrogen dioxide, SO_2_: Sulfur dioxide, O_3_: Ozone

FigS9 BICs(A-G) and AOEs(a-g) of the fitted models for meteorological factors and air pollutants effects for different dfs of the chosen ns. PM_2.5_: Particulate matter with aerodynamic diameters≤2.5μm, PM_10_: Particulate matter with aerodynamic diameters≤10μm, NO_2_: Nitrogen dioxide, SO_2_: Sulfur dioxide, O_3_: Ozone

FigS10 Cumulative exposure-effects curves for DNLM models controlling for confounding variables. PM_2.5_: Particulate matter with aerodynamic diameters≤2.5μm, PM_10_: Particulate matter with aerodynamic diameters≤10μm, NO_2_: Nitrogen dioxide, SO_2_: Sulfur dioxide, O_3_: Ozone
